# A FtsZ Inhibitor That Can Utilize Siderophore-Ferric Iron Uptake Transporter Systems for Activity against Gram-Negative Bacterial Pathogens

**DOI:** 10.3390/antibiotics13030209

**Published:** 2024-02-22

**Authors:** Eric J. Bryan, Qi Qiao, Yuxuan Wang, Jacques Y. Roberge, Edmond J. LaVoie, Daniel S. Pilch

**Affiliations:** 1 Department of Pharmacology, Rutgers Robert Wood Johnson Medical School, Piscataway, NJ 08854, USA; ejb236@dls.rutgers.edu (E.J.B.); yw782@rwjms.rutgers.edu (Y.W.); 2 Department of Molecular Design and Synthesis, Rutgers University Biomedical Innovation Cores, Piscataway, NJ 08854, USA; qq10@research.rutgers.edu (Q.Q.); jr1257@research.rutgers.edu (J.Y.R.); 3 Department of Medicinal Chemistry, Ernest Mario School of Pharmacy, Rutgers, The State University of New Jersey, Piscataway, NJ 08854, USA; elavoie@pharmacy.rutgers.edu

**Keywords:** *Klebsiella pneumoniae*, *Acinetobacter baumannii*, antibiotic-siderophore conjugate, antibiotic uptake, FtsZ inhibitor-antibiotic synergy

## Abstract

The global threat of multidrug-resistant Gram-negative bacterial pathogens necessitates the development of new and effective antibiotics. FtsZ is an essential and highly conserved cytoskeletal protein that is an appealing antibacterial target for new antimicrobial therapeutics. However, the effectiveness of FtsZ inhibitors against Gram-negative species has been limited due in part to poor intracellular accumulation. To address this limitation, we have designed a FtsZ inhibitor (**RUP4**) that incorporates a chlorocatechol siderophore functionality that can chelate ferric iron (Fe^3+^) and utilizes endogenous siderophore uptake pathways to facilitate entry into Gram-negative pathogens. We show that **RUP4** is active against both *Klebsiella pneumoniae* and *Acinetobacter baumannii*, with this activity being dependent on direct Fe^3+^ chelation and enhanced under Fe^3+^-limiting conditions. Genetic deletion studies in *K. pneumoniae* reveal that **RUP4** gains entry through the FepA and CirA outer membrane transporters and the FhuBC inner membrane transporter. We also show that **RUP4** exhibits bactericidal synergy against *K. pneumoniae* when combined with select antibiotics, with the strongest synergy observed with PBP2-targeting β-lactams or MreB inhibitors. In the aggregate, our studies indicate that incorporation of Fe^3+^-chelating moieties into FtsZ inhibitors is an appealing design strategy for enhancing activity against Gram-negative pathogens of global clinical significance.

## 1. Introduction

The global spread of multidrug-resistant (MDR) bacterial pathogens represents a public health crisis [1,2,3]. Patients diagnosed with MDR infections suffer much worse prognoses, including extended hospital stays and higher mortality rates, than patients with drug-susceptible infections [1]. Globally, 1.3 million deaths per year are directly attributable to resistant bacterial infections [4], with this figure predicted to rise to 10 million annual deaths by 2050 in the absence of intervention [5]. Of particular concern are ESKAPE pathogens [6], bacterial strains that are typically associated with high rates of clinical resistance [7,8]. Two of the more resistant ESKAPE pathogens are the Gram-negative species *Klebsiella pneumoniae* and *Acinetobacter baumannii*, each of which is responsible for more than 100,000 deaths annually [2,4]. Current clinical antibiotics, including carbapenems, third generation cephalosporins, and fluoroquinolones, are becoming increasingly ineffective against these two pathogens, leaving clinicians with few treatment options [4,9,10].

The threat posed by highly resistant Gram-negative pathogens emphasizes the need to develop new and effective antibiotics with novel bacterial targets. One promising new antibacterial drug target is FtsZ, an essential and highly conserved bacterial divisome protein that is not targeted by any current clinical drug [11,12]. FtsZ is a GTPase that polymerizes into a structure known as the Z-ring at the midcell of a dividing bacterium [13]. The FtsZ Z-ring is a major factor in driving bacterial cytokinesis, acting as a scaffold for recruitment of essential cell division components, which include the cell wall-forming penicillin-binding proteins (PBPs) [11,14]. In addition, FtsZ acts in concert with the cell wall synthesis coordinator, MreB, in both septal cell wall synthesis and lateral cell wall synthesis at the poles of rod-shaped bacteria [15,16].

FtsZ is a highly druggable target, and benzamide-based FtsZ inhibitors (including PC190723, TXA707, and TXH9179, as well as their corresponding prodrugs TXY541, TXA709, and TXH1033, respectively) with potent bactericidal activity against clinically significant Gram-positive pathogens, such as methicillin- and vancomycin-resistant *S. aureus* (MRSA and VRSA, respectively), have been previously described by us and others [17,18,19,20,21,22,23,24,25,26,27,28,29,30,31]. However, these FtsZ-targeting drug candidates are typically associated with poor activity against Gram-negative bacteria [20,32]. One of the principal resistance mechanisms in Gram-negative bacteria is their outer membrane, a hydrophobic barrier that limits the intracellular accumulation of most antibiotics, including benzamide FtsZ inhibitors [33,34,35]. This barrier must be overcome to improve the intracellular uptake of FtsZ inhibitors by Gram-negative pathogens as well as their resultant antibacterial efficacy.

One appealing approach for enhancing antibiotic uptake by Gram-negative bacteria is the design of agents that incorporate iron-chelating siderophore functionalities into their structures [36]. Ferric iron (Fe^3+^) is an essential nutrient for bacterial growth and survival, providing a critical redox factor for many important metabolic processes, including respiration, oxygen transport, and DNA synthesis [37,38]. However, Fe^3+^ availability is typically limited at sites of infection, due to restriction by the innate immune system in host environments [37,38,39,40]. To facilitate the scavenging of Fe^3+^ from the environment, bacteria synthesize and export molecules, termed siderophores, which chelate Fe^3+^ and are then internalized by the bacteria via dedicated uptake pathways [41,42]. Many clinically important Gram-negative pathogens, including *K. pneumoniae* and *A. baumannii*, rely on siderophores for Fe^3+^ acquisition and virulence [43,44]. Incorporation of Fe^3+^-chelating siderophore functionalities in antibacterial drug structures offers the potential for drug uptake by endogenous siderophore uptake pathways. In support of this design approach, cefiderocol, a PBP-targeting cephalosporin analog containing an Fe^3+^-chelating chlorocatechol moiety, has been recently approved for the treatment of complicated urinary tract infections caused by highly resistant Gram-negative pathogens [45,46,47].

**Figure 1:**
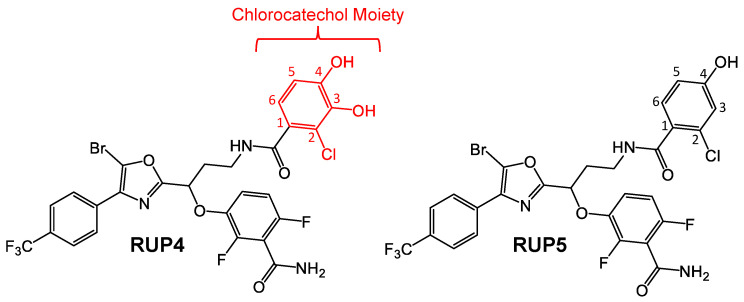
Chemical structures of **RUP4** and **RUP5**. The chlorocatechol siderophore moiety in **RUP4** is shown in red. The atomic numbering for the chlorocatechol moiety of **RUP4** and the equivalent ring of **RUP5** is also indicated.

In this study, we describe a novel oxazole-benzamide FtsZ inhibitor (**RUP4**) that, like cefiderocol, incorporates a Fe^3+^-chelating chlorocatechol moiety. This chlorocatechol moiety contains hydroxyl groups at the 3- and 4-positions (Figure 1), which are designed to chelate Fe^3+^. Significantly, **RUP4** is active against both *K. pneumoniae* and *A. baumannii*, in marked contrast to a corresponding negative control compound (**RUP5**) that lacks the hydroxyl group at the 3-position of the catechol moiety (Figure 1). We show that the antibacterial activity of **RUP4** against *K. pneumoniae* is due to its ability to chelate Fe^3+^ and that the compound utilizes endogenous *K. pneumoniae* siderophore-Fe^3+^ uptake transporters for entry into the bacteria. We also show that **RUP4** acts synergistically against *K. pneumoniae* when combined with select β-lactam antibiotics as well as a MreB inhibitor, with this bactericidal synergy being greatest upon combination with the PBP2-targeting antibiotic mecillinam and the MreB inhibitor TXH11106. Viewed as a whole, our results highlight **RUP4** as an appealing proof-of-concept agent for the design of next-generation benzamide FtsZ inhibitors with enhanced activity against Gram-negative bacterial pathogens.

## 2. Results

### 2.1. Evaluation of **RUP4** and **RUP5** for Their Ability to Chelate Fe^3+^

**Figure 2:**
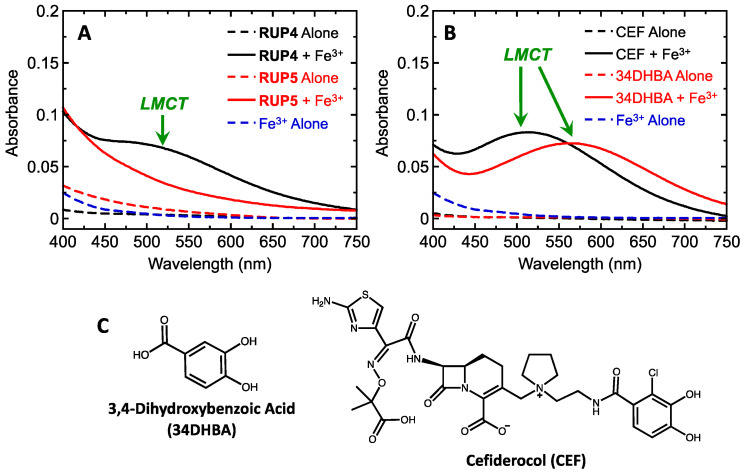
Absorption spectra of **RUP4** and **RUP5** (**A**) or 3,4-dihydroxybenzoic acid (34DHBA) and cefiderocol (CEF) (**B**) in the absence or presence of an equimolar concentration of added Fe^3+^. All compounds were used at a concentration of 50 µM, and all absorbance spectra were acquired in 75 mM Tris-HCl (pH 8.0). The green arrows in (**A**) and (**B**) highlight absorption peaks representative of ligand-to-metal charge transfer (LMCT) reactions associated with compound chelation of Fe^3+^. The chemical structures of 34DHBA and CEF are shown in (**C**).

We first sought to determine the potential for **RUP4** and **RUP5** to chelate Fe^3+^. Nolan and co-workers have shown that catechol chelation of Fe^3+^ is associated with a ligand-to-metal charge transfer (LMCT) reaction that is manifested by the induction of a peak in the absorption spectrum of the catechol in the wavelength region of 400–750 nm [48]. In this connection, we measured the absorption spectra of 50 µM **RUP4** and **RUP5** in the absence or presence of an equimolar concentration of Fe^3+^. The addition of Fe^3+^ to **RUP4** resulted in the induction of a broad absorbance peak in the region between 400 and 750 nm that was absent from the spectrum of either **RUP4** or Fe^3+^ alone (Figure 2A). This induced absorption peak is indicative of an LMCT reaction (denoted by the green arrow in Figure 2A). We observed the induction of similar LMCT peaks in the absorption spectra of our positive comparator control agents 3,4-dihydroxybenzoic acid (34DHBA) and cefiderocol (CEF) upon addition of Fe^3+^ (see spectra in Figure 2B and chemical structures of 34DHBA and CEF in Figure 2C). Note that the LMCT peak in the spectrum of **RUP5** in the presence of Fe^3+^ is markedly reduced in magnitude compared with the corresponding LMCT peak in the spectrum of **RUP4** (Figure 2A).

### 2.2. Evaluation of **RUP4** and **RUP5** for Antibacterial Activity against K. pneumoniae and A. baumannii

**Figure 3:**
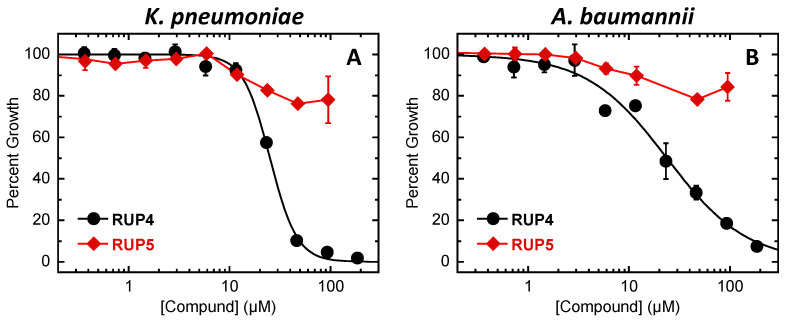
Impact of increasing concentrations of **RUP4** or **RUP5** on the percent growth of *K. pneumoniae* 10031 (**A**) or *A. baumannii* 19606 (**B**) in modified M9 media. Each experimental data point represents the average of three replicates, and the error bars reflect the standard deviation from the mean. The black curves represent nonlinear least squares fits of the experimental data points for **RUP4** using Equation (1).

We characterized the antibacterial activity of **RUP4** and **RUP5** against *K. pneumoniae* (ATCC 10031) and *A. baumannii* (ATCC 19606) in Fe^3+^-limiting M9 media. In these studies, both compounds were assessed at concentrations up to their limits of solubility, with the resulting growth profiles being shown in Figure 3. **RUP4** inhibits the growth of both *K. pneumoniae* (Figure 3A) and *A. baumannii* (Figure 3B) with similar IC_50_ values of 25 ± 1 and 24 ± 2 µM, respectively, as well as corresponding minimal inhibitory concentration (MIC) values of 46 µM against *K. pneumoniae* and 185 µM against *A. baumannii* (Table 1). We also determined the minimal bactericidal concentration (MBC) of **RUP4** against *K. pneumoniae* to be 92 µM, a value twice the corresponding MIC of 46 µM, with the MBC of **RUP4** against *A. baumannii* being greater than the solubility limit of the compound (>185 µM). In marked contrast to **RUP4**, **RUP5** did not inhibit the growth of *K. pneumoniae* or *A. baumannii* to any significant degree (Figure 3), with associated IC_50_, MIC, and MBC values all being greater than the solubility limit of the compound (>95 µM) (Table 1).

### 2.3. Impact of Added Exogenous Fe^3+^ on the Antibacterial Activity of **RUP4** against K. pneumoniae

**Figure 4:**
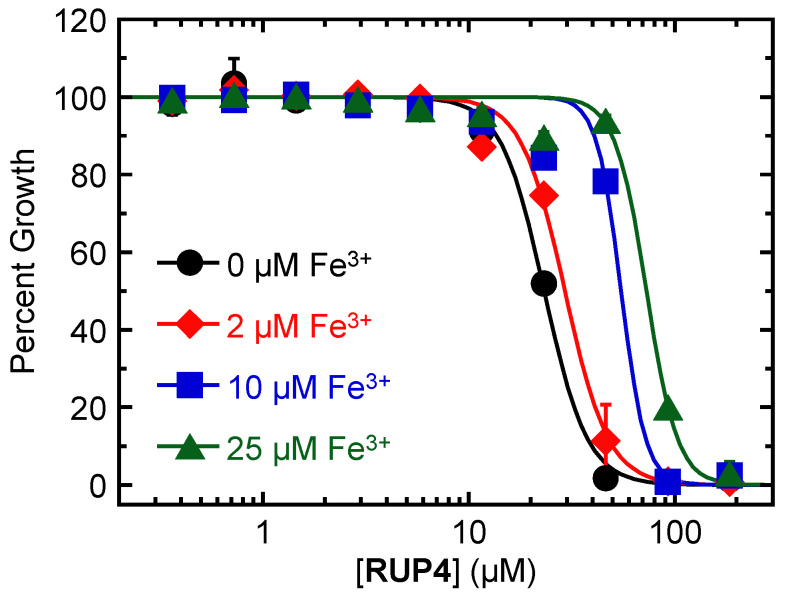
Impact of increasing concentrations of added exogenous Fe^3+^ on the antibacterial activity of **RUP4** against *K. pneumoniae* 10031 in modified M9 media. The media was supplemented with 0, 2, 10, or 25 µM Fe^3+^. Each experimental data point represents the average of three replicates, and the error bars reflect the standard deviation from the mean. The solid curves represent nonlinear least squares fits of the experimental data points using Equation (1).

We sought to determine whether the observed activity of **RUP4** against *K. pneumoniae* is sensitive to the concentration of exogenous Fe^3+^. To this end, we explored the impact of adding 0, 2, 10, or 25 µM exogenous Fe^3+^ on the activity of **RUP4** against *K. pneumoniae* in modified M9 media. The resulting growth profiles are shown in Figure 4. Significantly, the addition of exogenous Fe^3+^ markedly reduced the activity of **RUP4**, as reflected by IC_50_ values that increased with increasing concentrations of added exogenous Fe^3+^ [with these IC_50_ values being 23 ± 1 µM with 0 µM added Fe^3+^, 29 ± 1 µM with 2 µM added Fe^3+^, 55 ± 6 µM with 10 µM added Fe^3+^, and 73 ± 4 µM with 25 µM added Fe^3+^] (Table 2).

### 2.4. Expression of Siderophore Biosynthesis, Exporter, and Uptake Transporter Genes by K. pneumoniae in Nutrient-Rich CAMH Media Versus Fe^3+^-Limiting M9 Media

As an initial step toward determining whether **RUP4** can utilize the native siderophore-Fe^3+^ uptake machinery of target bacteria, we first explored how Fe^3+^ restriction affects the expression of native siderophore uptake, biosynthesis, and exporter genes in *K. pneumoniae*. To this end, we used RT-qPCR to compare the expression of key siderophore uptake, biosynthesis, and exporter genes in nutrient-rich CAMH media versus Fe^3+^-limiting M9 media. For siderophore uptake transporter genes, we evaluated the expression of the enterobactin outer membrane transporter *fepA*, the catecholate outer membrane transporters *cirA* and *fiu*, the ferrichrome outer membrane transporter *fhuA*, the enterobactin ABC inner membrane transporter components *fepD* and *fepG* (channel components) as well as *fepC* (ATPase), and the ferrichrome ABC inner membrane transporter components *fhuB* (channel) and *fhuC* (ATPase). For siderophore biosynthesis genes, we evaluated the expression of the catecholate synthesis pathway component *entB* and the enterobactin synthesis pathway component *entF*. For siderophore exporter genes, we evaluated expression of the enterobactin exporter *entS*. The expression of the bacterial transcription factor *rho* was used as an endogenous control for all comparisons.

**Figure 5:**
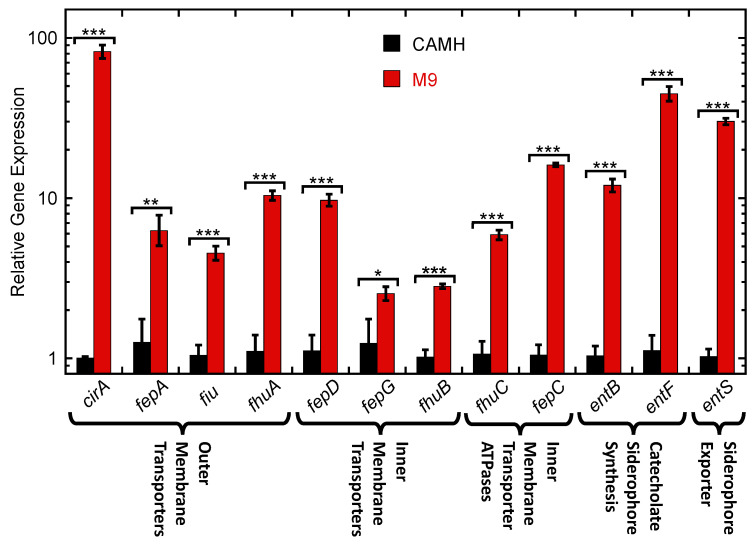
Relative expression of Fe^3+^ uptake outer membrane transporter, inner membrane transporter, inner membrane transporter ATPase, catecholate siderophore synthesis, and siderophore exporter genes in *K. pneumoniae* 10031 cells grown in either cation-adjusted Mueller Hinton (CAMH) or modified M9 media. Each bar represents an average of five replicates, and the error bars reflect the standard error from the mean. The statistical differences between gene expression levels in CAMH and M9 were determined by a Student’s *t*-test. ***, *p* ≤ 0.001; **, 0.01 ≥ *p* > 0.001; *, 0.05 ≥ *p* > 0.01.

Expression of all siderophore genes was significantly upregulated in M9 media relative to CAMH media (Figure 5). Among the outer membrane uptake transporters, *cirA* was upregulated to the greatest extent (82.1-fold), followed by *fhuA*, *fepA*, and *fiu* at 10.4-, 6.3-, and 4.5-fold, respectively. Among the inner membrane uptake transporters, *fepD* was upregulated to the greatest extent (9.7-fold), followed by *fhuB* and *fepG* at 2.8- and 2.5-fold, respectively. The inner membrane uptake transporter ATPases *fepC* and *fhuC* were upregulated by 16.1- and 5.9-fold, respectively. Finally, the catecholate siderophore synthesis genes *entF* and *entB* as well as the siderophore exporter gene *entS* were upregulated by 44.8-, 12.0-, and 30.1-fold, respectively.

### 2.5. Impact of Deleting Select Siderophore-Fe^3+^ Uptake Transporter Genes on **RUP4** Activity against K. pneumoniae

**Figure 6:**
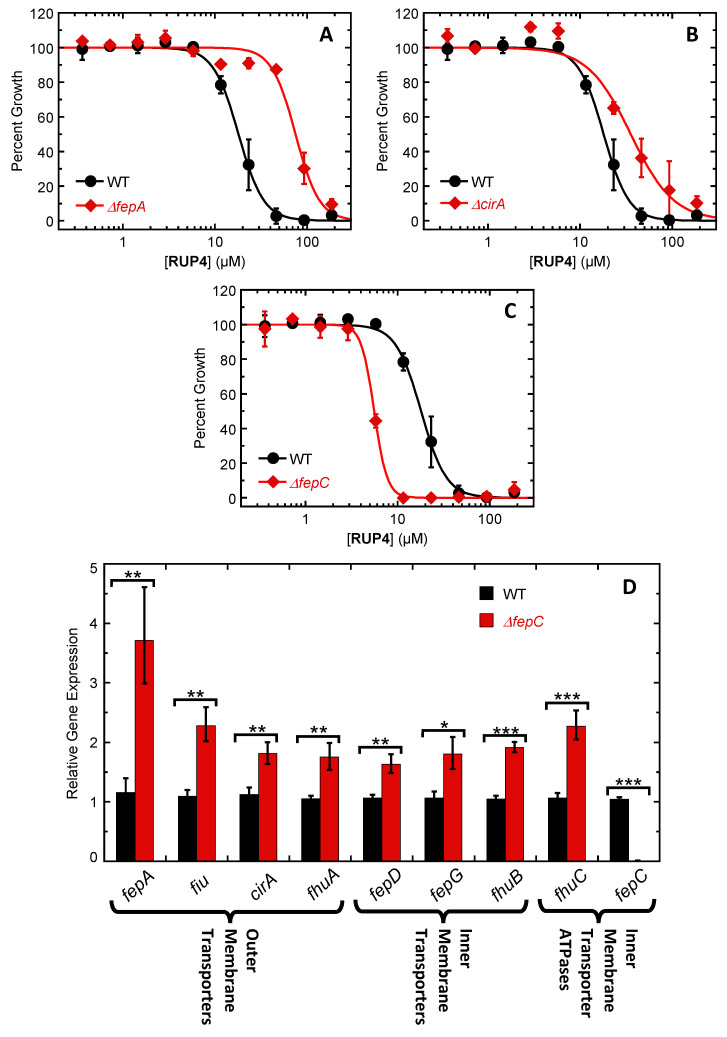
(**A**–**C**) Impact of deleting the *fepA* (**A**), *cirA* (**B**), or *fepC* (**C**) gene on the antibacterial activity of **RUP4** against *K. pneumoniae* 10031 in modified M9 media. Each experimental data point represents the average of five replicates, and the error bars reflect the standard error from the mean. The solid curves represent nonlinear least squares fits of the experimental data points using Equation (1). (**D**) Relative expression of Fe^3+^ uptake outer membrane transporter, inner membrane transporter, and inner membrane transporter ATPase genes in wild-type (WT) versus Δ*fepC K. pneumoniae* 10031 cells grown in modified M9 media. Each bar represents an average of five replicates, and the error bars reflect the standard error from the mean. The statistical differences between relative gene expression levels in WT versus Δ*fepC* cells were determined by a Student’s *t*-test. ***, *p* ≤ 0.001; **, 0.01 ≥ *p* > 0.001; *, 0.05 ≥ *p* > 0.01.

Towards the goal of elucidating the specific uptake pathway of **RUP4**, we generated mutant strains of *K. pneumoniae* containing a deletion of the siderophore-Fe^3+^ outer membrane transporter *fepA*, *fiu*, or *cirA*. We then compared the activity of **RUP4** against each of these deletion mutant strains (∆*fepA*, ∆*fiu*, or ∆*cirA*) relative to the activity against the wild-type (WT) *K. pneumoniae* strain, with the corresponding growth profiles comparing ∆*fepA* and ∆*cirA* versus WT being shown in Figure 6A. The activity of **RUP4** was attenuated by approximately 4-fold against the ∆*fepA* mutant and 2-fold against the ∆*cirA* mutant, as reflected by IC_50_ values of 75 ± 5 µM for the ∆*fepA* strain and 35 ± 5 µM for the ∆*cirA* strain relative to 18 ± 1 µM for WT (Table 3). By contrast, the activity of **RUP4** against the ∆*fiu* strain (IC_50_ = 22 ± 4 µM) was comparable to the activity versus WT (Table 3).

We also generated a mutant strain of *K. pneumoniae* containing a deletion of the siderophore-Fe^3+^ inner membrane transporter ATPase *fepC*, with the corresponding growth profiles comparing Δ*fepC* versus WT being shown in Figure 6C. Unlike the attenuated activity exhibited by **RUP4** versus the Δ*fepA* and Δ*cirA* mutant strains relative to WT, the corresponding activity of the compound versus the Δ*fepC* mutant strain was enhanced by approximately 3-fold (IC_50_ = 5.6 ± 0.1 µM) relative to WT (Figure 6C, Table 3). To further explore the basis for the increased activity of **RUP4** against the Δ*fepC* mutant, we used RT-qPCR to compare the expression of the siderophore outer membrane uptake genes *fepA*, *fiu*, *cirA*, and *fhuA* as well as the inner membrane uptake genes *fepD*, *fepG*, *fhuB*, and *fhuC* in the Δ*fepC* mutant strain relative to WT. The expression of all the tested genes was significantly upregulated (Figure 6D). Among the outer membrane uptake genes, expression of *fepA* was upregulated to the greatest extent (3.7-fold), followed by *fiu*, *cirA*, and *fhuA* at 2.3-, 1.8-, and 1.7-fold, respectively. Expression of the inner membrane uptake genes *fepD*, *fepG*, *fhuB*, and *fhuC* was upregulated by 1.6-, 1.8-, 1.9-, and 2.3-fold, respectively.

### 2.6. Direct Binding of **RUP4** to Purified K. pneumoniae FtsZ (KpFtsZ)

**Figure 7:**
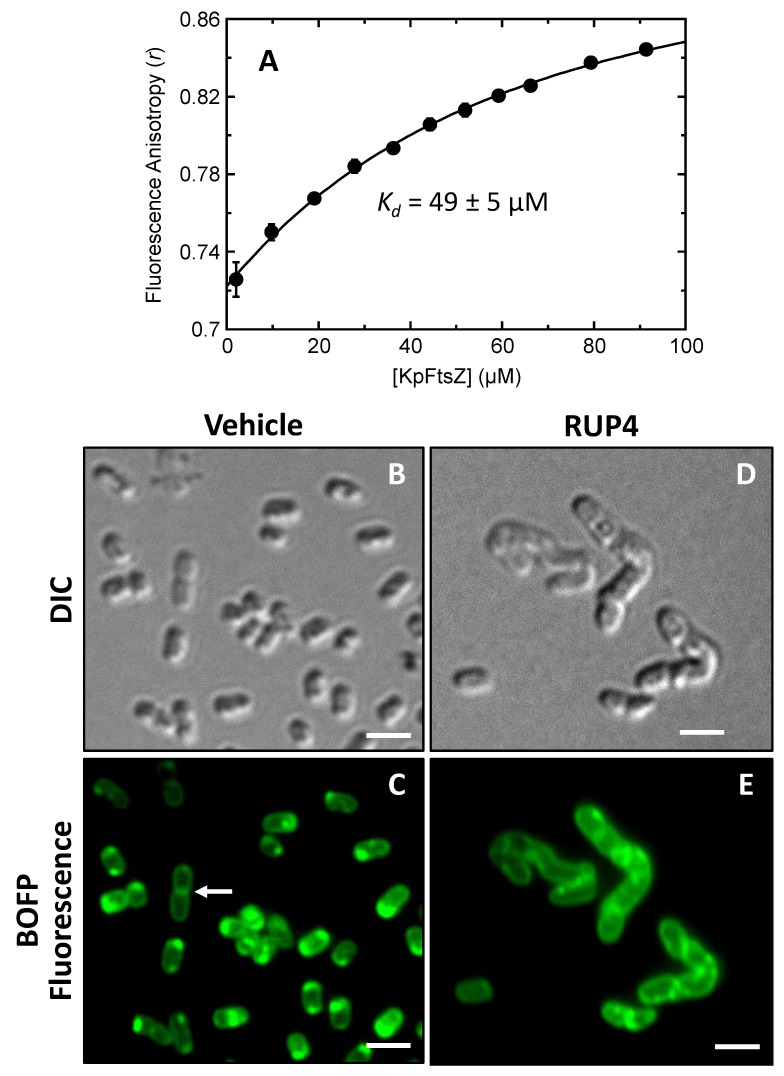
(**A**) Fluorescence anisotropy (*r*) of 20 µM RUP4 as a function of increasing concentrations of *K. pneumoniae* FtsZ (KpFtsZ). The titration experiment was conducted at 25 °C in a solution containing 50 mM Tris-HCl (pH 7.6) and 50 mM KCl. The solid curve represents a nonlinear least squares fit of the experimental data points using Equation (2), with the indicated dissociation constant (*K_d_*) value being derived from this fit. (**B**–**E**) Differential interference contrast (DIC) and fluorescence micrographs of *K. pneumoniae* 10031 cells treated for 3 h with either DMSO vehicle (**B**,**C**) or 185.3 µM (4× MIC) **RUP4** (**D**,**E**). The fluorescence micrographs in (**C**,**E**) depict cells labeled with BOFP just prior to visualization. The white arrow in (**C**) highlights an actively dividing cell with FtsZ localized to the septum at midcell. Scale bars reflect 1 µm.

As an initial step towards the validation of FtsZ as the antibacterial target of **RUP4**, we sought to demonstrate that **RUP4** can directly interact with purified KpFtsZ. To this end, we leveraged the intrinsic fluorescence properties of **RUP4**, whose excitation and emission spectra are shown in Figure S1. Specifically, we monitored the impact of added KpFtsZ on the fluorescence anisotropy of **RUP4** at 25 °C. The addition of KpFtsZ induced an increase in fluorescence anisotropy with increasing protein concentration (Figure 7A), indicative of a direct binding reaction. The resulting binding profile was fit with Equation (2) to yield a *K_d_* value of 49 ± 5 µM.

### 2.7. Impact of **RUP4** on Cell Division and FtsZ Localization in K. pneumoniae

To further establish FtsZ as the antibacterial target of **RUP4**, we explored the impact of **RUP4** treatment on cell morphology and FtsZ localization in *K. pneumoniae* grown in Fe^3+^-limiting M9 media. In these studies, *K. pneumoniae* cells were treated for 3 h with either DMSO vehicle or **RUP4** at 185.3 µM (4× MIC) and then labeled with 0.1 µM BOFP. Differential interference contrast (DIC) and fluorescence micrographs of the treated cells are shown in Figure 7B–E. Treatment with vehicle results in normal cell morphology and size, with approximately 3–8% of cells actively undergoing cell division. An actively dividing cell with FtsZ localized to the septum at the midcell is highlighted by the white arrow in Figure 7C. In non-dividing vehicle-treated cells, FtsZ often appears to localize in punctate regions at the cell poles (Figure 7C). In contrast to vehicle treatment, **RUP4** treatment results in enlarged and elongated cells with no defined septa and FtsZ more diffusely localized to foci throughout the cell as well as along the cell periphery (Figure 7D,E). This pattern of behavior is similar to that previously observed in rod-shaped bacteria that have been treated with a FtsZ inhibitor or in which FtsZ has been depleted [20,49,50].

### 2.8. Bactericidal Synergy of **RUP4** in Combination with Select PBP-Targeting β-Lactam Antibiotics and an MreB-Targeting Agent against K. pneumoniae

**Figure 8:**
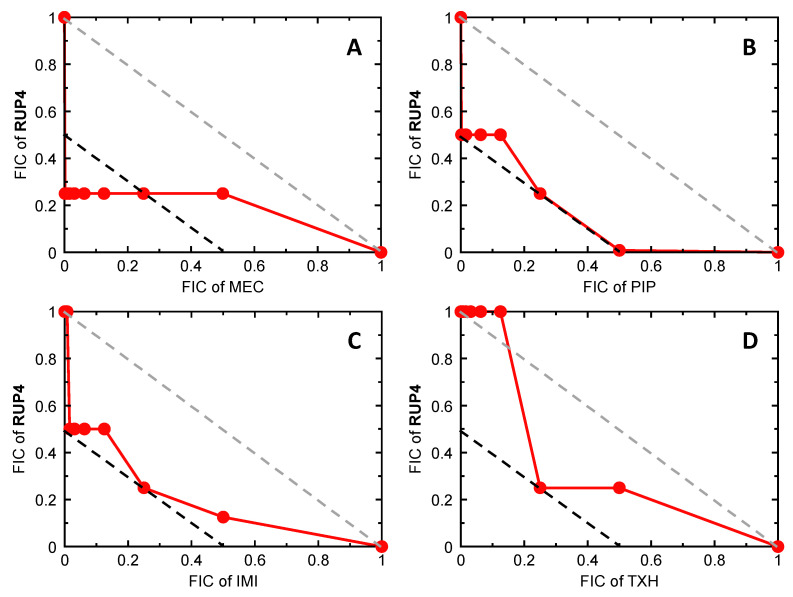
Isobolograms for *K. pneumoniae* 10031 treated with a combination of **RUP4** and either mecillinam (MEC) (**A**), piperacillin (PIP) (**B**), imipenem (IMI) (**C**), or TXH11106 (TXH) (**D**). In each plot, the black dashed line indicates the upper boundary for a synergistic combination, while the gray dashed line indicates the upper boundary for an additive combination. FIC denotes the fractional inhibitory concentration, as defined in Materials and Methods (Section 4.8.1.).

We assessed the potential of **RUP4** to act synergistically in combination with various PBP-targeting β-lactam antibiotics [mecillinam (MEC), amoxicillin (AMX), piperacillin (PIP), cefazolin (CFZ), imipenem (IMI), and meropenem (MER)] as well as an MreB-targeting agent TXH11106 (TXH) we have previously developed [51]. In this connection, we evaluated the intrinsic activities of the test agents against *K. pneumoniae*, with the resulting MIC values being listed in Table S1. We then used a checkerboard assay to define the fractional inhibitory concentration (FIC) of each test agent and **RUP4** when used in combination against *K. pneumoniae*. For each combination, synergy is indicated when the FIC values for both agents are ≤0.25. Figure 8 shows the isobolograms plotting the FICs for **RUP4** as a function of the FICs for MEC (Figure 8A), PIP (Figure 8B), IMI (Figure 8C), or TXH (Figure 8D). All four test agents act synergistically with **RUP4**, as indicated by the presence of combination FICs in the lower left quadrant of the isobolograms (on or to the left of the black dashed line in each plot). The corresponding isobolograms for the combinations of **RUP4** and CFZ, AMX, or MER are plotted in Figure S2, with these plots revealing additive behavior (with combination FICs falling to the right of the black dashed line and on or to the left of the gray dashed line).

Another indication of synergistic versus additive activity is provided by the FIC index (FICI), defined as the sum of the FICs of the two agents being tested in combination. A FICI ≤ 0.5 is indicative of synergy, while a FICI > 0.5 but ≤1 is reflective of additivity. The synergistic actions of **RUP4** in combination with MEC are reflected by a FICI of 0.251 (Table 4), with those of **RUP4** in combination with PIP, IMI, or TXH being reflected by FICI values of 0.5. The additive behavior of **RUP4** in combination with CFZ, AMX, or MER is reflected by FICI values of 0.508, 0.531, and 0.531, respectively (Table 4).

**Figure 9:**
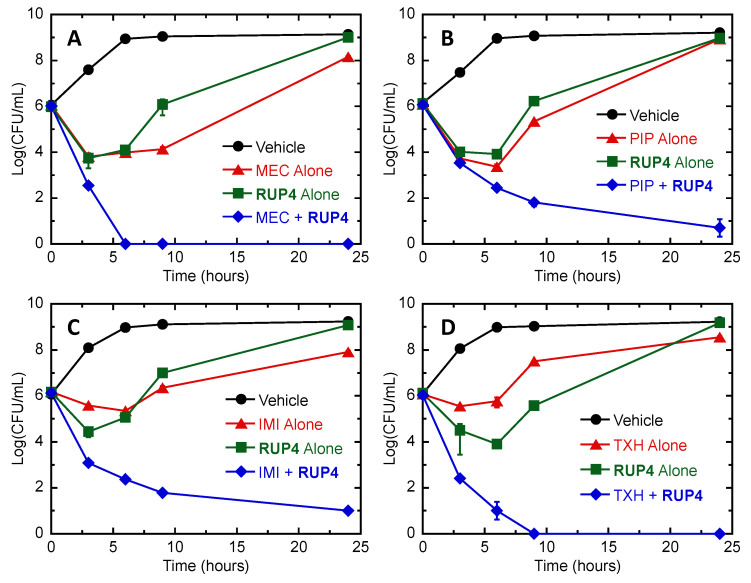
Time–kill curves for *K. pneumoniae* 10031 showing bactericidal synergy between **RUP4** and mecillinam (MEC) (**A**), piperacillin (PIP) (**B**), imipenem (IMI) (**C**), or TXH11106 (TXH) (**D**). Bacteria were treated with DMSO vehicle (black), test agent alone at 0.5× MIC (red), **RUP4** alone at 0.5× MIC (green), or a combination of test agent at 0.5× MIC and **RUP4** at 0.5× MIC (blue). Each experimental data point represents the average of two replicates, with the error bars reflecting the standard deviation from the mean.

In addition to checkerboard assays for synergy, we also performed time–kill assays of **RUP4** in combination with MEC, PIP, IMI, or TXH against *K. pneumoniae*. In these assays, cells were treated with DMSO vehicle, 0.5× MIC test agent alone, 0.5× MIC **RUP4** alone, or a combination of 0.5× MIC test agent and 0.5× MIC **RUP4,** and the number of colony-forming units (CFUs) was assessed after 0, 3, 6, 9, and 24 h of treatment. The resulting time–kill curves are shown in Figure 9. Treatment with either the test agent alone or **RUP4** alone resulted in growth at 24 h comparable to that of the vehicle. By contrast, the combination of each test agent with **RUP4** resulted in bactericidal behavior. In this connection, complete kill was observed after 6 h of treatment with the combination of MEC and **RUP4**, and after 9 h of treatment with the combination of TXH and **RUP4** (Figure 9A,D). Combination of **RUP4** and IMI or PIP resulted in 5-logs of kill within 24 h of treatment (Figure 9B,C).

## 3. Discussion

Gram-negative bacterial pathogens are often more difficult to treat than Gram-positive pathogens due to the presence of an outer membrane barrier that limits the intracellular accumulation of many antibiotics [33,35]. In this connection, benzamide-based FtsZ inhibitors have been associated with potent activity against Gram-positive pathogens like *S. aureus* while exhibiting poor or no activity against Gram-negative pathogens [17,18,19,20,21,22,23,24,25,26,27,28,29,30,31,34]. Here, we describe a strategy for overcoming this limitation by developing an oxazole-benzamide FtsZ inhibitor (**RUP4**) that incorporates a chlorocatechol siderophore moiety (Figure 1) and can utilize endogenous siderophore-Fe^3+^ uptake pathways to facilitate entry into the target bacterial cells. Significantly, **RUP4** is active against both *K. pneumoniae* and *A. baumannii* (Figure 3, Table 1), two Gram-negative pathogens of acute clinical importance. As indicated by MIC, the observed activity of **RUP4** is approximately 4-fold greater against *K. pneumoniae* (MIC = 46.3 µM) than against *A. baumannii* (MIC = 185.3 µM), though **RUP4** exhibited a similar IC_50_ value of approximately 25 µM against both pathogens (Table 1).

### 3.1. Importance of the Catechol Siderophore Functionality for the Antibacterial Activity of **RUP4**

Our design strategy is premised on the hypothesis that the catechol siderophore moiety plays an important role in promoting the observed antibacterial activity of **RUP4**. To this end, we first sought to establish that the two hydroxyl groups at positions 3 and 4 of the **RUP4** catechol functionality (Figure 1) can chelate Fe^3+^. Catechol chelation of Fe^3+^ is associated with a ligand-to-metal charge transfer (LMCT) reaction that is measurable by the induction of an absorption peak between 400 and 750 nm [48]. Consistent with such an Fe^3+^ chelation reaction, **RUP4** exhibits a robust LMCT peak in the presence of Fe^3+^ that is absent in the absorption spectra of either **RUP4** or Fe^3+^ alone (Figure 2A). The Fe^3+^-chelating capability of **RUP4** is further supported by the corresponding spectra of the positive control molecules cefiderocol and 34DHBA, which exhibit similar LMCT peaks in the presence of Fe^3+^ (Figure 2B). By contrast, the spectrum of the negative control compound **RUP5**, which lacks the 3-hydroxyl on the catechol ring, has a markedly reduced LMCT peak (Figure 2A), indicative of an attenuated ability to chelate Fe^3+^.

Armed with the knowledge that **RUP4** chelates Fe^3+^ to a significantly greater extent than **RUP5**, we determined if this differential capability manifested in a corresponding difference in antibacterial activity. Significantly, in striking contrast to **RUP4**, **RUP5** exerts a minimal impact on the growth of either *K. pneumoniae* or *A. baumannii* at concentrations up to its solubility limit of approximately 95 µM (Figure 3, Table 1). The absence of significant **RUP5** activity implies that the antibacterial activity of **RUP4** is linked to its ability to chelate Fe^3+^. Consistent with this notion, we show that the activity of **RUP4** against *K. pneumoniae* is systematically reduced in the presence of increasing concentrations of added exogenous Fe^3+^ (Figure 4), with IC_50_ values ranging from 23 µM in the absence of added Fe^3+^ to 73 µM in the presence of 25 µM added Fe^3+^ (Table 2). In addition, the expression of siderophore-Fe^3+^ uptake transporter, catecholate siderophore biosynthesis, and siderophore exporter genes in *K. pneumoniae* is significantly upregulated under Fe^3+^-limiting conditions (Figure 5). These collective results are indicative of **RUP4** utilizing endogenous siderophore-Fe^3+^ uptake pathways for its activity against *K. pneumoniae*.

**Figure 10:**
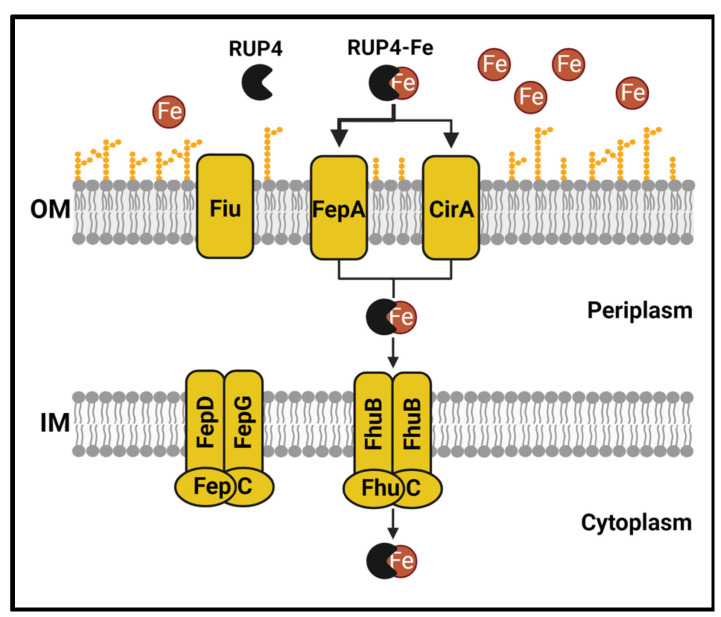
Putative mechanism for the uptake of **RUP4** into *K. pneumoniae* by endogenous outer membrane (OM) and inner membrane (IM) siderophore-Fe^3+^ uptake transporters.

We next sought to determine the specific siderophore-Fe^3+^ uptake transporters that **RUP4** can utilize for entry into *K. pneumoniae*. To this end, we generated deletion mutant strains of three different outer membrane transporters (Δ*fepA*, Δ*cirA*, and Δ*fiu*) in *K. pneumoniae* 10031 and compared the activity of **RUP4** against each deletion mutant strain relative to WT. The activity of **RUP4** was reduced against both the Δ*fepA* and Δ*cirA* strains compared to WT (Figure 6A,B), with the extent of this reduction being approximately 4-fold for the Δ*fepA* strain and 2-fold for the Δ*cirA* strain, as reflected by IC_50_ values (Table 3). By contrast, **RUP4** exhibited similar activity against the Δ*fiu* strain relative to WT (Table 3). These results suggest that **RUP4** crosses the outer membrane through both the FepA and CirA transporters, with FepA being the predominant transporter (schematically depicted in Figure 10).

We also generated a deletion mutant strain of the inner membrane transporter ATPase *fepC*. Surprisingly, the activity of **RUP4** against the Δ*fepC* strain was improved by approximately 3-fold compared to WT (Figure 6C, Table 3), suggesting that the enterobactin inner membrane transporter FepCDG is not the principal transporter through which **RUP4** crosses the inner membrane. To better understand the enhanced activity of **RUP4** against the Δ*fepC* strain, we measured the expression of all siderophore-Fe^3+^ uptake transporter genes in the Δ*fepC* strain relative to WT. Significantly, expression of the outer membrane transporter genes *fepA* and *fiu,* as well as the ferrichrome inner membrane transporter genes *fhuB* and *fhuC,* was among the most upregulated (Figure 6D), with *fepA* expression being upregulated to the greatest extent of all (approximately 3.7-fold). As previously noted, FepA appears to be the principle outer membrane transporter utilized by **RUP4**. Furthermore, aside from FepCDG, FhuBC is the only other inner membrane uptake transporter present in *K. pneumoniae* 10031. These collective results suggest that **RUP4** utilizes the FhuBC transporter for crossing the inner membrane, and the increased activity of **RUP4** against the Δ*fepC* strain relative to WT is primarily due to the increased expression of genes encoding the FepA and FhuBC transporters. Viewed as a whole, our genetic studies in *K. pneumoniae* are consistent with a putative uptake pathway (depicted in Figure 10) in which **RUP4** utilizes predominantly FepA but also CirA for crossing the outer membrane while utilizing FhuBC for crossing the inner membrane.

### 3.2. Validation of FtsZ as the Antibacterial Target of **RUP4**

As important as establishing the importance of the catechol siderophore moiety for the antibacterial activity of **RUP4** is verifying that incorporation of the catechol functionality does not hinder the ability of the compound to target FtsZ. In this connection, we first sought to confirm that **RUP4** can directly interact with FtsZ. To assay **RUP4** binding, we monitored changes in the fluorescence anisotropy of **RUP4** as a function of added KpFtsZ. We found that KpFtsZ increased the fluorescence anisotropy of **RUP4** in a concentration-dependent manner (Figure 7A), indicative of a direct binding reaction. Analysis of the resulting binding profile revealed that the binding interaction is associated with a *K_d_* of 49 µM, a value similar in magnitude to the observed IC_50_ (25 µM) and MIC (46 µM) of **RUP4** against wild-type *K. pneumoniae* (Table 1).

To further establish FtsZ as the antibacterial target of **RUP4**, we explored the impact of **RUP4** on morphology and FtsZ localization in live *K. pneumoniae* cells. Previous studies have shown that FtsZ inhibition or depletion in rod-shaped Gram-negative bacteria results in elongated cells that lack septa, with FtsZ being more diffusely localized throughout the elongated cell [49,50]. Consistent with this behavior, treatment of *K. pneumoniae* with **RUP4** results in elongated cells that lack septa and exhibit a pattern of dispersed FtsZ localization (Figure 7D,E). This behavior markedly contrasts that observed in vehicle-treated cells, which exhibit normal size and shape as well as FtsZ localization either to the poles of non-dividing cells or to the septa of actively dividing cells (Figure 7B,C).

A third observation consistent with FtsZ being the antibacterial target of **RUP4** is the bactericidal action of the compound, as revealed by an MBC/MIC ratio of 2 against *K. pneumoniae* (Table 1). This type of behavior agrees with numerous previous studies demonstrating that FtsZ inhibitors act in a bactericidal fashion [17,18,19,20,21,22,25,27,29]. In the aggregate, the collective studies described above are fully consistent with **RUP4** acting as a FtsZ inhibitor.

### 3.3. Synergistic Drug Combinations with **RUP4**

Synergistic antibiotic combinations offer the potential for enhancing potency, reducing the potential for both toxicity and resistance, and repurposing existing drugs [52]. We therefore explored if the activity of **RUP4** against *K. pneumoniae* could be enhanced by a synergistic combination with other antibiotics. To this end, we probed for potential synergy when combining **RUP4** with select β-lactam antibiotics as well as a MreB inhibitor. **RUP4** exhibited bactericidal synergy in combination with the PBP2-targeting antibiotic mecillinam, the PBP3-targeting antibiotic piperacillin, the carbapenem antibiotic imipenem (which primarily targets PBP2 and PBP4), and the MreB-targeting agent TXH11106 (Figure 8 and Figure 9, Table 4). The strongest bactericidal synergy was observed with mecillinam and TXH11106, with complete bacterial kill observed after 6–9 h of combination treatment (Figure 9A,D). Both PBP2 and MreB are critical components of the bacterial elongasome complex that synthesizes the lateral cell wall in rod-shaped bacteria and are known to transiently localize to the early divisome at the septum [15,53,54]. These results suggest that combining FtsZ inhibitors with agents that target the bacterial elongasome complex, including PBP2 and MreB, is an especially appealing strategy for enhancing the activity of FtsZ inhibitors against Gram-negative pathogens. In addition, mecillinam is associated with very poor intrinsic activity against *K. pneumoniae* 10031, with an associated MIC of 3147 µM (Table S3). However, combination with **RUP4** strongly activates mecillinam, as reflected by an FIC of 0.0005 (Table 4). Thus, combination with FtsZ inhibitors can also repurpose β-lactam antibiotics for use against Gram-negative pathogens that were previously resistant to those drugs. Future studies will be directed at further optimizing the antibacterial activity of **RUP4** both through chemical modification and in combination with other antibiotics.

## 4. Materials and Methods

### 4.1. Bacterial Strains, Growth Media, and Reagents

*K. pneumoniae* ATCC 10031 and *A. baumannii* ATCC 19606 were acquired from the American Type Culture Collection (ATCC, Manassas, VA, USA). Strains of *K. pneumoniae* 10031 containing the Δ*fepA*, Δ*fiu*, Δ*cirA*, and Δ*fepC* deletions were generated as described in the Supplementary Materials (Section 2). Unless otherwise noted, bacterial strains were grown in modified M9 minimal growth media (6.8 g/L Na_2_HPO_4_, 3 g/L KH_2_PO_4_, 0.5 g/L NaCl, 1 g/L NH_4_Cl) supplemented with 0.4% glucose, 2 mM MgSO_4_, 0.1 mM CaCl_2_, 0.2% low-iron casein amino acids, and 16.5 µg/mL thiamine hydrochloride [48]. Agar, casein amino acids, cation-adjusted Mueller-Hinton (CAMH) media, Luria-Bertani (LB) media, and trypticase soy agar (TSA) were obtained from Becton Dickinson (Franklin Lakes, NJ, USA). Phosphate-buffered saline (PBS) was obtained from Lonza (Walkersville, MD, USA), and Tris-acetate-EDTA (TAE) buffer was obtained from Thermo Fisher Scientific (Waltham, MA, USA. Imipenem was obtained from LKT Labs (St. Paul, MN, USA), mecillinam was obtained from RPI (Mount Prospect, IL, USA), and meropenem was obtained from Toku-E (Bellingham, WA, USA). Amoxicillin, piperacillin, cefazolin, pentamidine isethionate, D-(+)-glucose, and thiamine hydrochloride were obtained from Sigma-Aldrich (St. Louis, MO, USA).

### 4.2. Compound Synthesis and K. pneumoniae FtsZ Protein Expression and Purification

**RUP4** and **RUP5** were synthesized as detailed in the Supplementary Materials. BOFP was synthesized as described previously [49] and is also available from Millipore-Sigma (#SCT090). TXH11106 was synthesized as described previously [51]. *K. pneumoniae* FtsZ (KpFtsZ) was expressed and purified as described previously [49].

### 4.3. Antibacterial Assays

Two-fold serial dilutions of **RUP4** and **RUP5** were prepared in microtiter plates containing M9 media following Clinical and Laboratory Standards Institute (CLSI) protocols for broth microdilution assays [55]. The final volume in each well was 100 µL, and each test concentration was present in triplicate. Log-phase bacteria were added to the microtiter plates at a final concentration of 5 × 10^5^ CFU/mL. All plates were incubated with shaking at 37 °C for 18 h. Bacterial growth in each well was determined by measuring the optical density at 600 nm (OD_600_) using a VersaMax plate reader (Molecular Devices, San Jose, CA, USA). The percent growth of bacteria in each well corresponding to the associated OD_600_ value was then determined by normalization with the OD_600_ value associated with the bacterial growth in the absence of the test compound (100% growth) and the background value in M9 media alone (0% growth). These percent growth values were then plotted versus compound concentration (C), and IC_50_ values were derived from non-linear least squares fits of the resulting plots with the following relationship:

(1) $$Percent\;Growth=\frac{100}{1+{\left(\frac{C}{{IC}_{50}}\right)}^{m}}$$

In this relationship, *m* is the Hill slope.

Values of MBC for **RUP4** were determined by plating (in triplicate) on TSA plates 50 µL of solution from the wells corresponding to 1×, 2×, and 4× MIC. These plates were incubated at 37 °C for 24 h and counts of CFU/mL were then determined. MBC values were defined as the lowest compound concentration that yielded ≥3 logs of kill relative to the initial inoculum of 5 × 10^5^ CFU/mL.

For assays determining the effect of added exogenous Fe^3+^ on **RUP4** activity, separate tubes containing 5 mL of M9 media were supplemented with 0, 2, 10, or 25 µM FeCl_3_. The tubes were then inoculated with *K. pneumoniae* 10031 and incubated with shaking at 37 °C overnight. The overnight cultures were then diluted 1:10 in M9 media supplemented with the appropriate concentration of FeCl_3_ and incubated with shaking at 37 °C to mid-log phase. Microtiter plates containing two-fold serial dilutions of **RUP4** were prepared as described above. The M9 media in each plate was supplemented with 0, 2, 10, or 25 µM FeCl_3_. Each microtiter plate was then inoculated with 5 × 10^5^ CFU/mL of log-phase bacteria grown in the corresponding concentration of FeCl_3_, and the microtiter plates were then incubated with shaking at 37 °C for 18 h. Bacterial growth in each well was determined as described above.

### 4.4. Absorption Spectroscopy

All absorption experiments were conducted at 25 °C using an AVIV model 14DS spectrophotometer (Aviv Biomedical, Lakewood, NJ, USA) equipped with thermoelectric temperature control. Solutions containing 50 µM of either **RUP4**, **RUP5**, 3,4-dihydroxybenzoic acid (34DHBA), or cefiderocol (CEF) were prepared in 75 mM Tris-HCl (pH 8.0), and an absorbance spectrum of each solution was acquired from 750 to 400 nm in 1-nm increments (with an averaging time of 0.5 s for each reading). Furthermore, 50 µM of FeCl_3_ was added directly to each compound solution, and an absorbance spectrum was obtained as described above. Corresponding absorbance spectra of buffer alone and buffer with 50 µM FeCl_3_ were acquired as background controls.

### 4.5. Fluorescence Spectroscopy

Fluorescence spectroscopy experiments were conducted at 25 °C using an AVIV Model ATF105 spectrofluorometer (Aviv Biomedical, Lakewood, NJ, USA), with bandwidths set to 5 nm in both the excitation and emission directions. Excitation and emission spectra of 20 µM RUP4 (in buffer consisting of 50 mM Tris-HCl, pH 7.6, and 50 mM KCl) were acquired in 1-nm increments with an averaging time of 1 s for each reading. The excitation spectrum was acquired with the emission wavelength set at 508 nm, and the emission spectrum was acquired with the excitation wavelength set at 319 nm. For these experiments, a quartz cell (Hellma, Plainview, NY, USA) was used with a path length of 1 cm in both the excitation and emission directions.

For the fluorescence anisotropy measurements, the excitation and emission wavelengths were set to 319 and 508 nm, respectively. KpFtsZ was titrated into a solution containing 20 µM **RUP4** in the same buffer used for the acquisition of the excitation and emission spectra, with the final concentrations of KpFtsZ ranging from 2–91 µM. After each protein addition, the samples were equilibrated for 5 min, and the fluorescence anisotropy (*r*) was recorded, with each *r* value being the average of 5 readings. For these experiments, a quartz ultra-micro cell (Hellma, Plainview, NY, USA) was used with a 2 mm × 5 mm aperture and a 15 mm center height. The path lengths in the excitation and emission directions were 1 and 0.2 cm, respectively.

Values of *r* were plotted as a function of KpFtsZ concentration, and the *K_d_* value for the binding reaction was derived from the non-linear least squares fit of the resulting plot with the following 1:1 binding formalism:

(2) $$r=r_{0}+\left(\frac{r_{\infty }-r_{0}}{2{\left[P\right]}_{tot}}\right)\times \left({\left[C\right]}_{tot}+{\left[P\right]}_{tot}+K_{d}\right)-\sqrt{{\left({\left[C\right]}_{tot}+{\left[P\right]}_{tot}\;{+K}_{d}\right)}^{2}-4{\left[C\right]}_{tot}{\left[P\right]}_{tot}}$$

In this relationship, *r*_0_ is the fluorescence anisotropy in the absence of KpFtsZ, *r*_∞_ is the fluorescence anisotropy in the presence of an infinite concentration of KpFtsZ, [*C*]*_tot_* is the total concentration of **RUP4**, and [*P*]*_tot_* is the total concentration of KpFtsZ.

### 4.6. Differential Interference Contrast (DIC) and Fluorescence Microscopy

Log-phase *K. pneumoniae* 10031 bacteria were diluted to 0.1 OD_600_ and treated (with shaking) for 3 h at 37 °C with **RUP4** at 4× MIC (185 µM) or an equal volume of DMSO. The cells were then washed, labeled with BOFP, and imaged by DIC and fluorescence microscopy as described previously [49].

### 4.7. RNA Extraction and Reverse Transcription-Quantitative Polymerase Chain Reaction (RT-qPCR) Assays

RT-qPCR assays comparing expression levels of siderophore-Fe^3+^ uptake genes (*fepA*, *cirA*, *fiu*, *cirA*, *fhuA*, *fepD*, *fepG*, *fhuB*, *fepC*, and *fhuC*), enterobactin synthesis genes (*entB* and *entF*), and an enterobactin exporter gene (*entS*) in *K. pneumoniae* 10031 cells grown in either CAMH or M9 media were performed using a Luna Universal One-Step RT-qPCR Kit (New England Biolabs, Ipswich, MA, USA). The bacterial transcription termination factor gene *rho* was used as an endogenous control for all assays. Briefly, RNA was extracted from five different replicates of log-phase *K. pneumoniae* 10031 cells grown in either CAMH or M9 media using a RNeasy Mini Kit (Qiagen, Germantown, MD, USA), and contaminating DNA was removed using a TURBO DNA-free Kit (Thermo Fisher Scientific, Waltham, MA, USA). Samples containing 1× Luna Universal One-Step Reaction Mix, 1× Luna WarmStart RT Enzyme Mix, 0.4 µM forward primer, 0.4 µM reverse primer, and 25 ng of total RNA were prepared in nuclease-free water. The primers used for each gene are listed in Table S2. The samples were then transferred to a MicroAmp Optical 96-Well Reaction Plate (Applied Biosystems, Waltham, MA, USA). Each sample was added in triplicate, and the final volume in each reaction well was 20 µL. The plate was then affixed with a MicroAmp Optical Adhesive Film (Applied Biosystems, Waltham, MA, USA) and centrifuged at maximum speed in a VWR plate centrifuge for 2 min. The reaction plate was then inserted into a QuantStudio 5 real-time PCR system (Applied Biosystems, Waltham, MA, USA) affixed with a 96-well 0.2 mL block. A comparative C_T_ (ΔΔC_T_) experiment was run with the SYBR Green Reagents chemistry setting, utilizing the standard Luna Universal One-Step RT-qPCR thermocycling protocol. The protocol consisted of: (a) reverse transcription, 1 cycle of 55 °C for 1 min; (b) initial denaturation, 1 cycle of 95 °C for 1 min; (c) 40 cycles of denaturation at 95 °C for 10 s followed by extension at 60 °C for 30 s with plate read; and (d) 1 cycle of a three-step melt curve consisting of (i) 95 °C for 15 s, (ii) 60 °C for 1 min, and (iii) 95 °C for 15 s. The resulting amplification plots were visualized and analyzed using the QuantStudio Design and Analysis Software (v1.5.2). The statistical significance of differences in relative gene expression was analyzed with a Student’s *t*-test. Assays comparing the gene expression levels of *fepA*, *cirA*, *fiu*, *fhuA*, *fepD*, *fepG*, *fhuB*, *fepC*, and *fhuC* in WT versus Δ*fepC K. pneumoniae* 10031 cells were performed as described above.

### 4.8. Antibacterial Synergy Assays

#### 4.8.1. Checkerboard Assays

The checkerboard assay [56] was used to evaluate the synergy between **RUP4** and the test agents mecillinam, piperacillin, imipenem, TXH11106, amoxicillin, meropenem, and cefazolin against *K. pneumoniae* 10031. These assays were performed as described previously [57]. The fractional inhibitory concentration for each test agent (FIC_TA_) was determined from the ratio of the MIC of the test agent in combination with **RUP4** to the MIC of the test agent alone. The fractional inhibitory concentration for **RUP4** (FIC_RUP4_) was determined from the ratio of the MIC of **RUP4** in combination with each test agent to the MIC of **RUP4** alone. The values of FIC_TA_ and FIC_RUP4_ were then used to determine the corresponding FIC index (FICI) using the relationship FICI = FIC_TA_ + FIC_RUP4_. A FICI ≤ 0.5 is indicative of a synergistic RUP4-test agent combination, while a FICI > 0.5 but ≤1.0 is indicative of an additive combination.

#### 4.8.2. Time–Kill Assays

Overnight cultures of *K. pneumoniae* 10031 were diluted to 10^6^ CFU/mL in four separate culture tubes containing 5 mL of M9 media. DMSO vehicle was added to the first tube, **RUP4** at 0.5× MIC (23 µM) alone was added to the second tube, test agent alone at 0.5× MIC (1574 µM mecillinam, 7.4 µM piperacillin, 0.8 µM imipenem, or 16 µM TXH11106) was added to the third tube, and a combination of 0.5× MIC **RUP4** and 0.5× MIC test agent was added to the final tube. Time-dependent kill assays were then conducted at 37 °C over a period of 24 h, as described previously [57].

## 5. Conclusions

Here we describe **RUP4** as a novel oxazole-benzamide FtsZ inhibitor that incorporates a chlorocatechol siderophore moiety and exhibits antibacterial activity against the clinically important Gram-negative pathogens *K. pneumoniae* and *A. baumannii*. Fe^3+^ chelation and addback studies establish that the catechol siderophore functionality plays a key role in the activity of **RUP4**. In this connection, **RUP4** is able to utilize endogenous siderophore uptake transporters for entry into *K. pneumoniae*, crossing the bacterial outer membrane through the FepA and CirA transporters and the inner membrane through the FhuBC transporter system. We validate FtsZ as the antibacterial target of **RUP4** by demonstrating the direct interaction of the compound with KpFtsZ as well as the induction of changes in morphology and FtsZ localization in treated *K. pneumoniae* cells consistent with FtsZ inhibition. Finally, synergy studies reveal that combinations of **RUP4** with select PBP- and MreB-targeting agents are associated with bactericidal synergy against *K. pneumoniae*, with combinations involving agents that target PBP2 and MreB being the most synergistic. Viewed as a whole, our results highlight the incorporation of Fe^3+^-chelating siderophore moieties into FtsZ inhibitors as a promising design strategy for enhancing activity against Gram-negative pathogens of acute clinical significance.

## Figures and Tables

**Table 1:** Antibacterial activities of **RUP4** and **RUP5** against *K. pneumoniae* and *A. baumannii*.

Strain	MIC (µM)		MBC (µM)	IC_50_ (µM)	
	RUP4	RUP5	RUP4	RUP4 *	RUP5
*K. pneumoniae* 10031	46	>95	92	25 ± 1	>95
*A. baumannii* 19606	185	>95	>185	24 ± 2	>95

MIC and MBC values were determined as described in Materials and Methods Section 4.3. * IC_50_ values for **RUP4** were determined from fits of the growth profiles shown in Figure 3 using Equation (1), with the indicated uncertainties reflecting the standard deviation of the fitted curves from the experimental data.

**Table 2:** Impact of added exogenous Fe^3+^ on the activity of **RUP4** against *K. pneumoniae* 10031.

Added [Fe^3+^] (µM)	IC_50_ (µM)
0	23 ± 1
2	29 ± 1
10	55 ± 6
25	73 ± 4

IC_50_ values were determined from fits of the growth profiles shown in Figure 4 using Equation (1), with the indicated uncertainties reflecting the standard deviation of the fitted curves from the experimental data.

**Table 3:** Impact of deleting specific siderophore-Fe^3+^ uptake transporters on the activity of **RUP4** against *K. pneumoniae* 10031.

Strain	IC_50_ (µM)
WT	18 ± 1
Δ*fepA*	75 ± 5
Δ*cirA*	35 ± 5
Δ*fiu*	22 ± 4
Δ*fepC*	5.6 ± 0.1

IC_50_ values were determined from fits of growth profiles represented by those shown in Figure 6 using Equation (1), with the indicated uncertainties reflecting the standard deviation of the fitted curves from the experimental data.

**Table 4:** Combinatorial activities **RUP4** and select PBP-targeting β-lactam antibiotics or an MreB-targeting agent against *K. pneumoniae* 10031.

Test Agent (TA)	FIC_TA_	FIC_RUP4_	FICI
MEC	0.0005	0.25	0.2505
PIP	0.25	0.25	0.5
IMI	0.25	0.25	0.5
TXH	0.25	0.25	0.5
CFZ	0.008	0.5	0.508
AMX	0.031	0.5	0.531
MER	0.031	0.5	0.531

MEC = Mecillinam; PIP = Piperacillin; IMI = Imipenem; TXH = TXH11106; CFZ = Cefazolin; AMX = Amoxicillin; MER = Meropenem. FIC_TA_ and FIC_RUP4_ reflect the fractional inhibitory concentrations of the indicated antibacterial test agent and **RUP4**, respectively. The fractional inhibitory concentration index (FICI) reflects the sum of FIC_TA_ + FIC_RUP4_. Synergistic combinations are indicated by a FICI ≤ 0.5 (denoted in red), while additive combinations are indicated by a FICI > 0.5 but ≤1.

## Data Availability

Data are contained within the article and Supplementary Materials.

## Supplementary Materials

The following supporting information can be downloaded at: https://www.mdpi.com/article/10.3390/antibiotics13030209/s1. Figure S1: Excitation and emission spectra of **RUP4** at 25 °C; Figure S2: Isobolograms for *K. pneumoniae* treated with a combination of **RUP4** and either cefazolin (CFZ), amoxicillin (AMX), or meropenem (MER); Table S1: Intrinsic activities of test β-lactam antibiotics and a MreB-targeting agent against *K. pneumoniae*; Table S2: Sequences of the oligonucleotide primers used in the qPCR studies of the *K. pneumoniae* wild-type and deletion mutant strains; Table S3: Sequences of the oligonucleotide primers used for deleting and sequencing ferric iron transporter genes in *K. pneumoniae*; Supplementary Methods: Compound synthesis and generation of Δ*fepA*, Δ*fiu*, Δ*cirA*, and Δ*fepC* mutant strains of *K. pneumoniae*. References [24,58,59] are cited in the Supplementary Materials.
