# Supplementary material for: A FtsZ Inhibitor That Can Utilize Siderophore-Ferric Iron Uptake Transporter Systems for Activity against Gram-Negative Bacterial Pathogens
Source: Antibiotics (Basel). 2024 Feb 22;13(3):209. doi: 10.3390/antibiotics13030209 (PMC10967458; doi:10.3390/antibiotics13030209)
Supplement: Supplementary file 1 [file antibiotics-13-00209-s001.zip › antibiotics-2879315-supplementary.pdf]

Supplementary Materials

A FtsZ Inhibitor That Can Utilize Siderophore-Ferric Iron Uptake Transporter Systems for Activity Against Gram-Negative Bacterial Pathogens

Eric J. Bryan[1], Qi Qiao[2], Yuxuan Wang[1], Jacques Y. Roberge[2], Edmond J. LaVoie[3], and Daniel S. Pilch[1,*]

[1] Department of Pharmacology, Rutgers Robert Wood Johnson Medical School, Piscataway, NJ 08854, USA; ejb236@dls.rutgers.edu (E.J.B.); yw782@rwjms.rutgers.edu (Y.W.).

[2] Department of Molecular Design and Synthesis, Rutgers University Biomedical Innovation Cores, Piscataway, NJ 08854, USA; qq10@research.rutgers.edu (Q.Q.); jr1257@research.rutgers.edu (J.R.).

[3] Department of Medicinal Chemistry, Ernest Mario School of Pharmacy, Rutgers-The State University of New Jersey, Piscataway, New Jersey 08854, USA; elavoie@pharmacy.rutgers.edu (E.J.L.).

*Correspondence: pilchds@rwjms.rutgers.edu

Supplementary Figures and Tables:

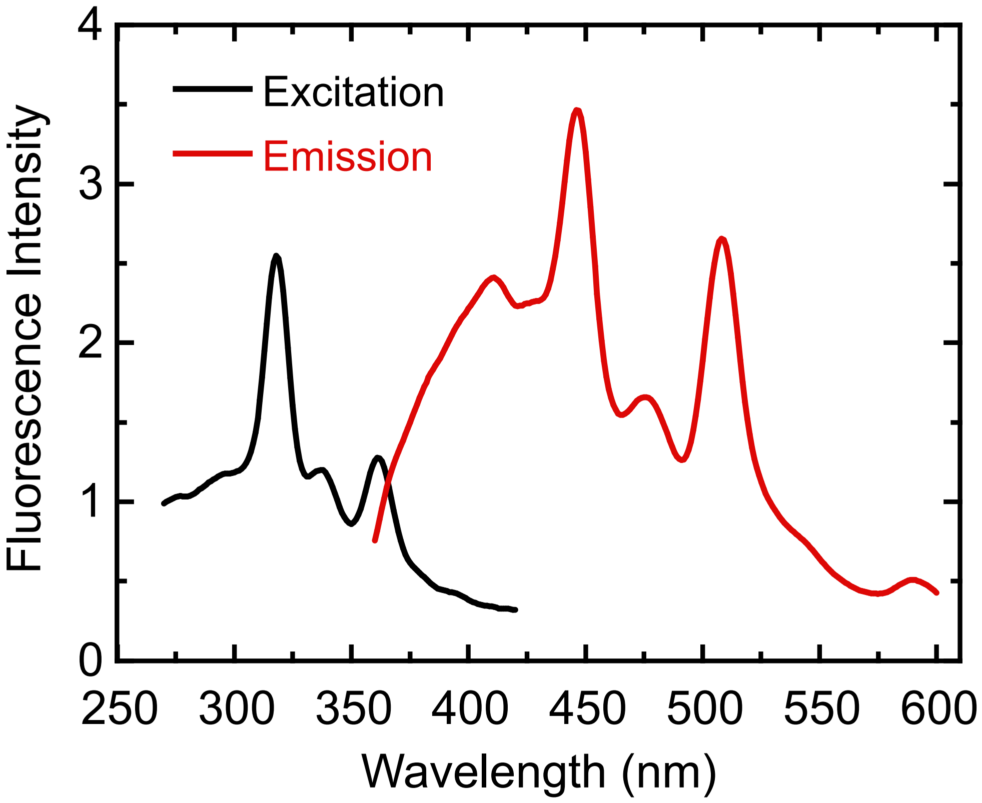

Figure S1: Fluorescence excitation (black) and emission (red) spectra of 20 μM RUP4 acquired at 25 °C in 50 mM Tris-HCl (pH 7.6) and 50 mM KCl. The excitation spectrum was acquired with the emission wavelength set at 508 nm, while the emission spectrum was acquired with the excitation wavelength set at 319 nm.

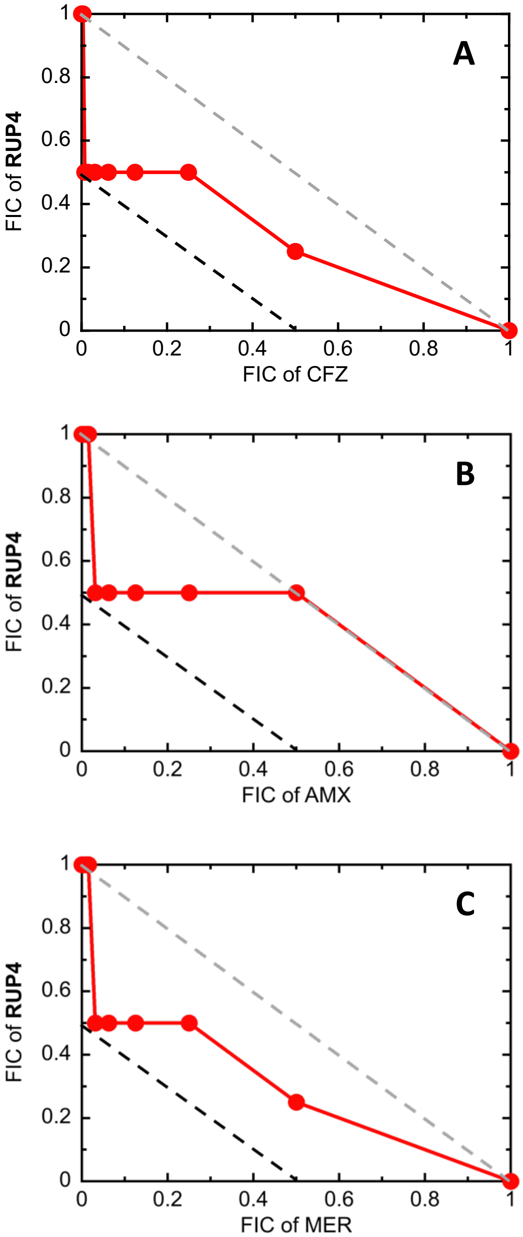

Figure S2: Isobolograms for *K. pneumoniae* 10031 treated with a combination of **RUP4** and either cefazolin (CFZ) (**A**), amoxicillin (AMX) (**B**), or meropenem (MER) (**C**). In each plot, the black dashed line indicates the upper boundary for a synergistic combination, while the gray dashed line indicates the upper boundary for an additive combination. FIC denotes the fractional inhibitory concentration, as defined in Materials and Methods Section 4.8.1.

Table S1. Intrinsic activities of test β-lactam antibiotics and a MreB-targeting agent against *K. pneumoniae* 10031.

Test Agent	MIC (μM)
MEC	3147
AMX	350
TXH	31
PIP	15
CFZ	4.2
IMI	1.6
MER	0.1

MEC = Mecillinam; AMX = Amoxicillin; TXH = TXH11106; PIP = Piperacillin; CFZ = Cefazolin; IMI = Imipenem; MER = Meropenem.

Table S2 Sequences of the oligonucleotide primers used in the qPCR studies of the *K. pneumoniae* 10031 wild-type and deletion mutant strains.

Name	Sequence (5′ → 3′)
KpFepA-qPCR-F	CGACTTAAAAGCCGAGACCAGC
KpFepA-qPCR-R	ATCTTGTTGCGGTAGTCGTTGC
KpFiu-qPCR-F	TGGCAAATCTTCGACAACCGTC
KpFiu-qPCR-R	GGAGAGTTCATACCCCTCCACG
KpCirA-qPCR-F	CATCAGCGTCATCACCCAACAG
KpCirA-qPCR-R	CTTGCGGTTATCCCCTTCGTTG
KpFhuA-qPCR-F	TCGTGGAAGGTATAATCCGCCC
KpFhuA-qPCR-R	ACGCCAATATTAACGTCACCGC
KpFepD-qPCR-F	TGCTATTACTCGCCTTAGCCGC
KpFepD-qPCR-R	CTTTGACAGGTTCCGCTGAAGG
KpFepG-qPCR-F	CGCCTGATCATTATCGGCATCG
KpFepG-qPCR-R	CCAGGTTTTACCCCAGGTCAGG
KpFhuB-qPCR-F	GAACATCATCATCCGTCCGCAC
KpFhuB-qPCR-R	CCGCATATGGTGATTTCAGGGC
KpFepC-qPCR-F	CTGCTGGAACTGTTGAGTGAGC
KpFepC-qPCR-R	CTGGTTAAGATCGTGCAGCACC
KpFhuC-qPCR-F	TCGGGATGCCGTAGATCTGTTC
KpFhuC-qPCR-R	GCGGAGAAATGATTGCTCAGGG
KpEntB-qPCR-F	CGCCCTGCTGAATGATATGTGG
KpEntB-qPCR-R	CCATTTCACCAGCACGGTATCG
KpEntF-qPCR-F	TACCTGTTCGGGTATTCGCTGG
KpEntF-qPCR-R	TTTCTCTGCCCAGTTTTGCGTC
KpEntS-qPCR-F	TGACCGCTATGAACGTAAACGC
KpEntS-qPCR-R	CAGATCCCCAGCAGGTAGATCG
KpRho-qPCR-F	GTACGCCGTCGCCAAAGATATC
KpRho-qPCR-R	CTGGAAAACCTGGCTCGTATGC

Table S3. Sequences of the oligonucleotide primers used for deleting and sequencing ferric iron transporter genes in *K. pneumoniae* 10031.

Name	Sequence (5′ → 3′)
Deletion Primers	
KpFepA-Del-F	CCCCTCCCGGTGGGAAGGGGCGTTTGGGGAACGTGGCGACAA AAAGCAGGAATAACAATG**ATTCCGGGGATCCGTCGACC**
KpFepA-Del-R	TTACCGCGGTTGCCCCCGCCCTTTGTCCAGCAAGGGCGGGGG AGGGAGAACCCGCTATCA**TGTAGGCTGGAGCTGCTTC**
KpFiu-Del-F	CGTCGCTTTTGCGCGGTGTGATATGTGCTCATAACAAAAAATA AGCCTGCATTTATCATG**ATTCCGGGGATCCGTCGACC**
KpFiu-Del-R	ATACATCATCGCGGTGTTCTCTCTAAAAAGTGGGGCGCAACG GCCCCACGCGCAGACTCA**TGTAGGCTGGAGCTGCTTC**
KpCirA-Del-F	GCGCGTGTGAGCAGCACCTTGTTGTTTGTTTGGTTGTACACCT CATGGAGATTTGGAATG**ATTCCGGGGATCCGTCGACC**
KpCirA-Del-R	GCGAAGCTGCTTCGCGGCGCATGTTCAGGCCTACAGGATGCG TTTTAGCGCCGGCAATCA**TGTAGGCTGGAGCTGCTTC**
KpFepC-Del-F	GTCAGCCTCGGCGGTATTTACCTTATTGCATTGTTGATTCAGG AGTCCCGCAAAAAATGA**ATTCCGGGGATCCGTCGACC**
KpFepC-Del-R	TGAGTTCGGGCTACAGCAGGTAGCCCGGCTCAGCGTAGCGCA AGCCGGGGAAAGAGGAGA**TGTAGGCTGGAGCTGCTTC**
Sequencing Primers	
KpFepA-Seq-F	GCTTTTGGCAACATTTATGCC
KpFepA-Seq-R	CGATCTGCTGGATGGTATAGC
KpFiu-Seq-F	GCAAGCCAGACGACGAATAAC
KpFiu-Seq-R	CGATAGCACGTCAGGAATGTG
KpCirA-Seq-F	CACATCGACGAGAAAACAGCG
KpCirA-Seq-R	AGCATCTCTTTGGCTAAACGC
KpFepC-Seq-F	CGGCTGTTTATGCCGTGG
KpFepC-Seq-R	GTTACCCCAGAATCTCCCTGA
Apra-Seq-R	CGAGACACTGCACCATTCTTC

The bold and underlined sequence reflects the FRT-apramycin-FRT complementary sequence either on the 5′ end of forward (F) primers or the 3′ end of reverse (R) primers.

Supplementary Methods:

1. Synthetic Methods

1.1. General Methods

All reagents were purchased and used without additional purifications. LC-MS was performed on a Shimadzu system: LC-MS 2010A using a 10 µL injection on Sunfire C18 column (3.5 µm, 4.6 x 50 mm) at a temperature of 40 °C with a 4-minute gradient from 5% B to 100% B (solvent A: 0.1% TFA in water; solvent B: 0.1% TFA in acetonitrile) and a flow rate of 2 mL/min. The detection used dual channel UV at 220 and 254 nm. Mass detection CDL voltage was -10 V. Alternatively, LC-MS was performed on an Agilent 1100 system with a Waters Micromass ZQ spectrometer using a 5 µL injection on an XBridge C18 (3.5 µm, 4.6 x 50 mm) column at a temperature of 40 °C with a 3-minute gradient from 5% B to 95% B (solvent A: 10 mM ammonium formate in water; solvent B: acetonitrile) and a flow rate of 2 mL/min. The detection used diode array scanning from 190 to 600 nm or with dual wavelength detectors at 220 and 254 nm. Mass detection cone voltage was 30 V. NMR spectra were obtained on a Varian VNMRS 500 MHz or a Bruker 400 MHz NMR in chloroform-d (^{1}H d 7.26; ^{13}C d 77.00), DMSO-d6 (^{1}H d 2.50; ^{13}C d 39.52) or methanol-d4 (^{1}H d 3.31; ^{13}C d 49.00). HRMS analyses were performed using a Bruker Apex 7T FTMS, with an ESI ion source in positive mode. Capillary exit voltage was 330 V. Microwave reactions were run on a Biotage Initiator system. Preparative HPLC was performed using a Phenomenex Gemini-NX C18 (3 µm, 75 x 30 mm) column with a 6-minute gradient from 1% B to 100% B (solvents A and B as described above) or on an ACCQPreP HP125 using a Waters XBridge BEH C18 (10 µm, 100 x 30 mm) column with an 8-minute gradient from 1% B to 100% B (solvents A and B as described above).

1.2. Synthesis of Requisite Intermediates

*1.2.1. 3-bromo-3-{5-bromo-4-[4-(trifluoromethyl)phenyl]oxazol-2-yl}propyl acetate (**I-1**) and 3-{5-bromo-4-[4-(trifluoromethyl)phenyl]oxazol-2-yl}-3-(3-carbamoyl-2,4-difluorophenoxy)propyl acetate (**I-2**).*

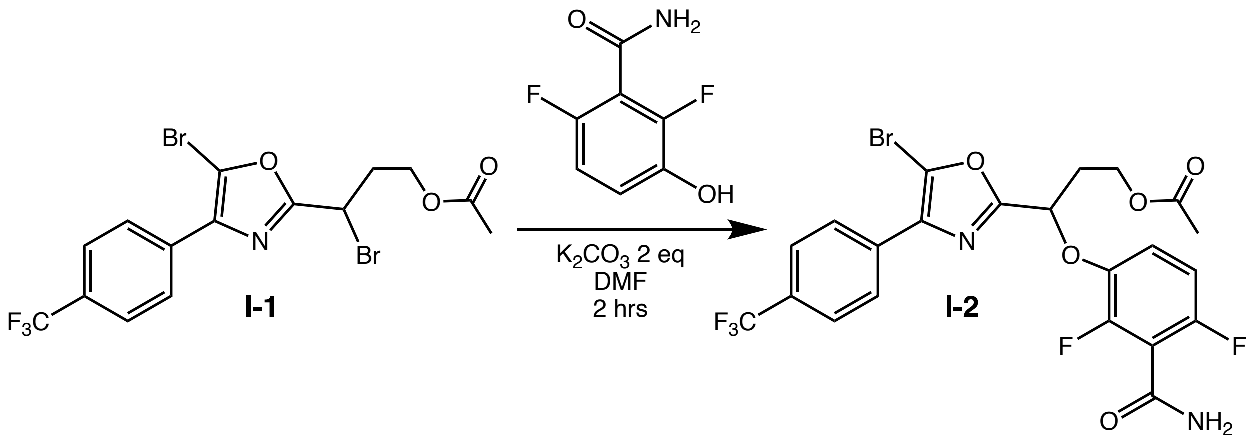

I-1 was prepared in 8 steps from the 4-trifluoromethylacetophenone as described previously [24]. To a solution of **I-1** (60 mg, 0.13 mmol, 1 eq) in DMF (4 mL), 2,6-difluoro-3-hydroxybenzamide (24.26 mg, 0.14 mmol, 1.1 eq) was added, followed by potassium carbonate (35.21 mg, 0.25 mmol, 2 eq). The reaction was stirred at room temperature for 2 hours. The reaction was diluted with EtOAc (100 mL), washed with water (50 mL), brine (50 mL), dried with Na_2SO_4, filtered, and concentrated under reduced pressure to give an oil. The crude product was purified by flash chromatography, eluting with 60% EtOAc/hexanes. The fractions containing product (**I-2**) were concentrated under reduced pressure to afford a colorless oil (22 mg, 30.7% yield). ^{1}H NMR (500 MHz, chloroform-d) δ 8.09 – 8.03 (m, 2H), 7.70 (ddt, J = 8.1, 1.3, 0.7 Hz, 2H), 7.16 (td, J = 9.1, 5.2 Hz, 1H), 6.86 (td, J = 9.1, 2.0 Hz, 1H), 5.95 (d, J = 39.9 Hz, 2H), 5.34 (dd, J = 8.5, 5.1 Hz, 1H), 4.44 – 4.29 (m, 2H), 2.66 – 2.55 (m, 1H), 2.46 (ddt, J = 14.7, 8.6, 5.2 Hz, 1H), 2.03 (s, 3H).

1.2.2. 3-{1-(5-bromo-4-[4-(trifluoromethyl)phenyl]oxazol-2-yl)-3-hydroxypropoxy}-2,6-difluorobenzamide ***(I-3)***.

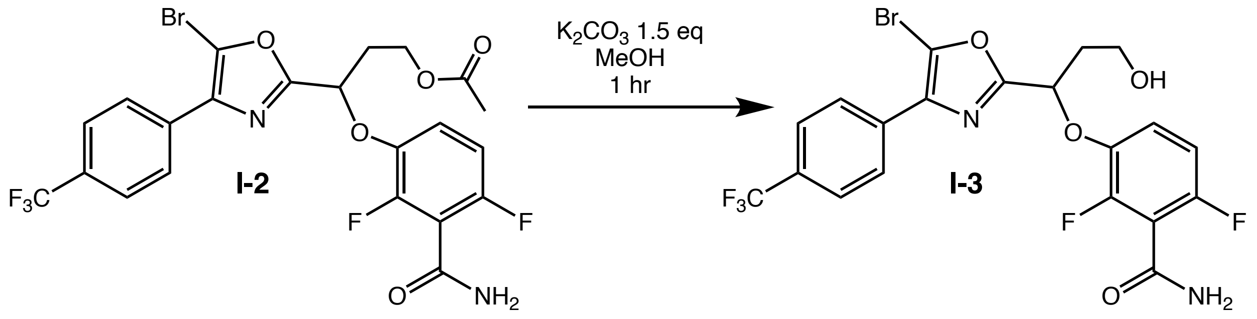

To a solution of **I-2** (34 mg, 0.06 mmol, 1 eq) in MeOH (5 mL), anhydrous potassium carbonate (12.54 mg, 0.09 mmol, 1.5 eq) was added and stirred for 1 hour. At completion, the reaction was diluted with water (30 mL), extracted twice with EtOAc (100 mL). The combined organic layers were dried over anhydrous Na_2SO_4, filtered, and concentrated under reduced pressure to give a crude oil. The crude product was purified by flash chromatography on silica gel, eluting with 60% EtOAc/hexanes. The fractions containing product (**I-3**) were concentrated under reduced pressure to afford an oil (31.5 mg, 57.9% yield). ^{1}H NMR (500 MHz, chloroform-d) δ 8.09 – 8.02 (m, 2H), 7.72 – 7.65 (m, 2H), 7.20 (td, J = 9.1, 5.2 Hz, 1H), 6.85 (td, J = 9.1, 1.9 Hz, 1H), 6.19 (d, J = 48.5 Hz, 2H), 5.47 (dd, J = 8.5, 5.0 Hz, 1H), 4.00 (ddd, J = 10.9, 8.4, 3.9 Hz, 1H), 3.89 (ddd, J = 10.8, 5.6, 4.6 Hz, 1H), 2.50 (dddd, J = 14.3, 8.5, 5.6, 3.9 Hz, 1H), 2.35 (ddt, J = 14.5, 8.4, 4.9 Hz, 1H), 2.19 (br. s, 1H). HRMS, theoretical m/z: 521.0130 MH^+, observed m/z: 521.0134 MH^+.

*1.2.3. 3-{5-bromo-4-[4-(trifluoromethyl)phenyl]oxazole-2-yl}-3-(3-carbamoyl-2,4-difluorophenoxy)propyl methanesulfonate (**I-4**), 3-(1-{5-bromo-4-[4-(trifluoromethyl)phenyl]furan-2-yl}-3-(2lambda4-triaza-1,2-dien-1-yl)propoxy)-2,6-difluorobenzamide (**I-5**), and 3-(3-amino-1-{5-bromo-4-[4-(trifluoromethyl)phenyl]oxazol-2-yl}propoxy)-2,6-difluorobenzamide (**I-6**).*

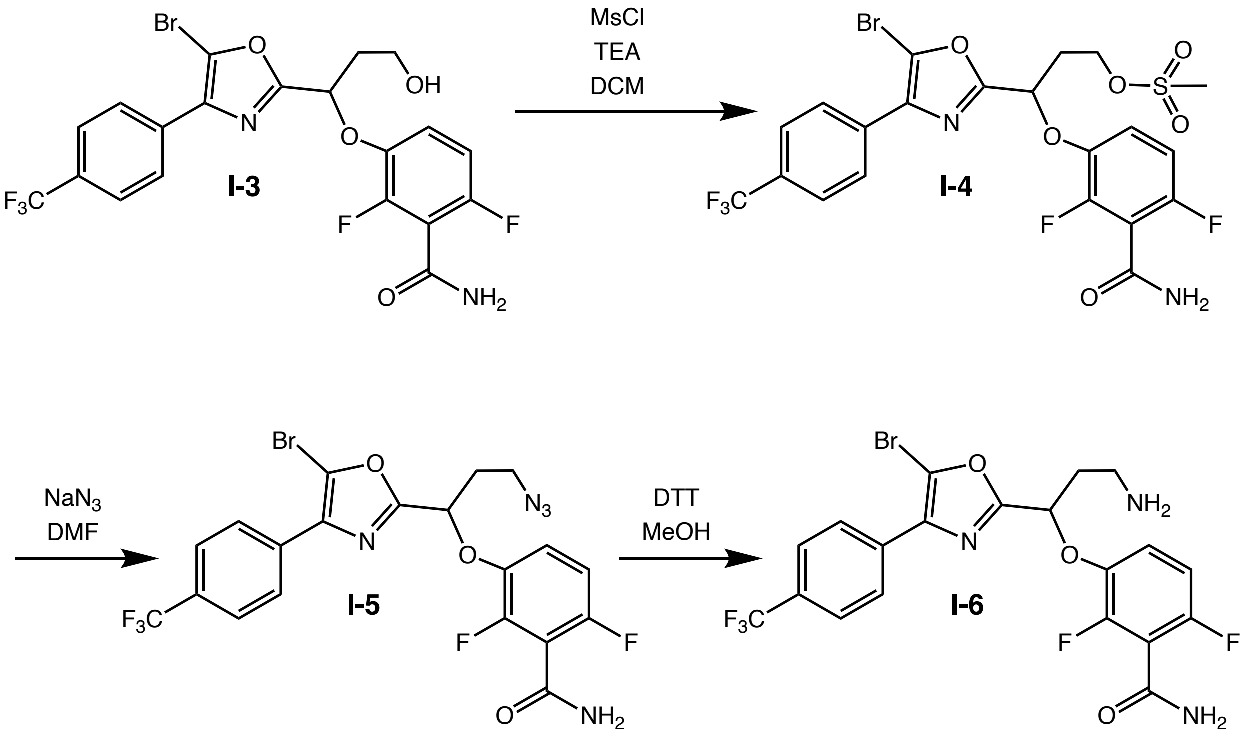

To a solution of **I-3** (28.3 mg, 0.05 mmol, 1 eq) in anhydrous THF (4 mL), TEA (15 µL, 0.11 mmol, 2 eq) was added, followed by MsCl (5 µL, 0.07 mmol, 1.2 eq). The reaction was stirred at room temperature for 1 hour. At completion, the reaction was diluted with water (30 mL), extracted twice with DCM (100 mL). The combined organic layers were then dried over Na_2SO_4. Concentration gave an oil (**I-4**), which was used in next step without purification (34.5 mg, 100% yield). ^1H NMR (500 MHz, Chloroform-d) δ 8.06 (ddt, J = 8.4, 1.6, 0.9 Hz, 2H), 7.70 (ddt, J = 8.3, 1.5, 0.8 Hz, 2H), 7.22 (td, J = 9.1, 5.1 Hz, 1H), 6.88 (td, J = 9.1, 1.9 Hz, 1H), 6.15 (d, J = 18.9 Hz, 2H), 5.39 (dd, J = 9.2, 4.3 Hz, 1H), 4.65 (ddd, J = 10.4, 9.2, 4.0 Hz, 1H), 4.50 (dt, J = 10.2, 4.9 Hz, 1H), 3.03 (s, 3H), 2.77 – 2.67 (m, 1H), 2.62 – 2.53 (m, 1H).

To a solution of **I-4** (420 mg, 0.70 mmol, 1 eq) in DMF (10 mL), NaN_3 (139 mg, 2.1 mmol, 3 eq) was added. The reaction was stirred at 50 °C for 3 hours. At completion, the reaction was diluted with water (30 mL), extracted twice with EtOAc (150 mL). The combined EtOAc layers were dried over anhydrous Na_2SO_4 and concentrated under reduced pressure to give an oil. The crude product was purified by flash chromatography on silica gel, eluting with 50% EtOAc/hexanes to afford a colorless oil (302 mg, 78.7% yield, **I-5**). ^{1}H NMR (500 MHz, Chloroform-*d*) δ 8.11 – 8.04 (m, 2H), 7.73 – 7.66 (m, 2H), 7.18 (td, J = 9.1, 5.2 Hz, 1H), 6.88 (td, J = 9.1, 2.0 Hz, 1H), 5.96 (d, J = 27.5 Hz, 2H), 5.34 (dd, J = 8.8, 4.8 Hz, 1H), 3.77 – 3.60 (m, 2H), 2.60 – 2.47 (m, 1H), 2.33 (dddd, J = 14.6, 8.7, 6.0, 4.8 Hz, 1H).

To a solution of **I-5** (50 mg, 0.09 mmol, 1 eq) in MeOH (3 mL), Et_3N (111 mg, 1.1 mmol, 152 µL, 12 eq) was added followed by dithiothreitol (169 mg, 1.1 mmol, 12 eq) [58]. The reaction was stirred at room temperature for 3 hours. At completion, the solution was diluted with MeOH (1 mL) and purified by preparatory chromatography, eluting with 1-100% CH_3CN/H_2O + 0.1% TFA. The fractions containing the desired product were concentrated to afford a white solid (34.8 mg, 73.2% yield, **I-6**). ^{1}H NMR (500 MHz, methanol-d4) δ 8.14 (d, J = 8.2 Hz, 2H), 7.77 (d, J = 8.3 Hz, 2H), 7.29 (td, J = 9.2, 5.2 Hz, 1H), 6.99 (td, J = 9.0, 1.9 Hz, 1H), 5.59 (dd, J = 8.0, 4.7 Hz, 1H), 3.39 – 3.33 (m, 1H), 3.30 – 3.25 (m, 1H), 2.69 – 2.57 (m, 1H), 2.50 (ddt, J = 14.4, 9.7, 5.3 Hz, 1H).. HRMS, theoretical m/z: 520.0290 MH^+, observed m/z: 520.0286 MH^+.

*1.3. Synthesis of **RUP4** and **RUP5***

*1.3.1. N-(3-{5-bromo-4-[4-(trifluoromethyl)phenyl]oxazol-2-yl}-3-(3-carbamoyl-2,4-difluorophenoxy)propyl)-2-chloro-3,4-dihydroxybenzamide (**RUP4**).*

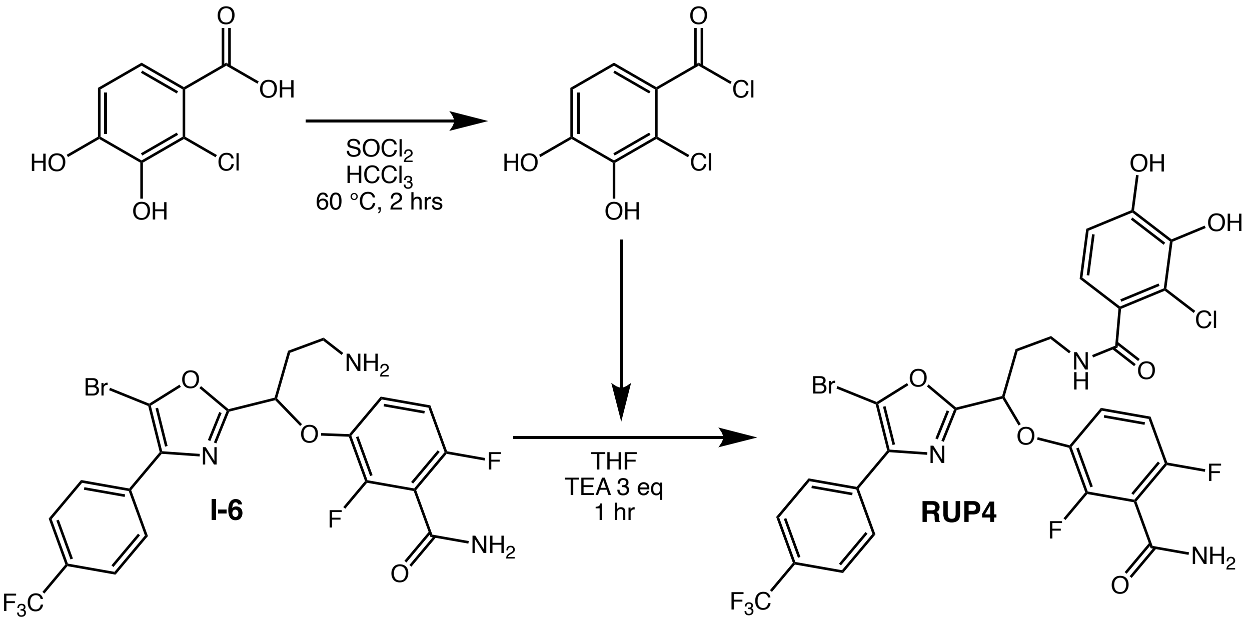

To a solution of 2-chloro-3,4-dihydroxybenzoic acid (7.5 mg, 0.04 mmol) in chloroform (0.5 mL), $SOCl_2$ (0.003 mL, 0.04 mmol) was added. The reaction was heated at 60 °C for 2 hours. After cooling to room temperature, the solvent was removed under reduced pressure and further dried on high vacuum for 3 hours. The acid chloride residue was used in next step without purification.

To a solution of **I-6** (19 mg, 0.04 mmol, 1 eq) in THF (2 mL), TEA (15 µL, 11 mg, 3 eq) was added followed by 2-chloro-3,4-dihydroxybenzoyl chloride (9 mg, 0.04 mmol, 1.2 eq) in THF (1 mL). The reaction was stirred at room temperature for 1 hour. Upon completion, the reaction was diluted with EtOAc (100 mL), washed with H_2O (35 mL), brine (35 mL), and dried over anhydrous Na_2SO_4. The solution was concentrated under reduced pressure to give an oil. The crude product was purified by preparative HPLC, eluting with 1-100% CH_3CN/H_2O + 0.1% TFA. The fractions containing the desired product were concentrated under reduced pressure to afford a white solid (6.4 mg, 25.4% yield, **RUP4**). ^1H NMR (500 MHz, methanol-d4) δ 8.51 (s, 4H), 8.18 – 8.06 (m, 2H),

7.80 – 7.71 (m, 2H), 7.33 (td, J = 9.2, 5.2 Hz, 1H), 7.02 – 6.93 (m, 1H), 6.81 (d, J = 8.3 Hz, 1H), 6.72 (d, J = 8.3 Hz, 1H), 5.54 (dd, J = 8.0, 5.3 Hz, 1H), 3.79 – 3.69 (m, 1H), 3.59 (dt, J = 13.7, 6.1 Hz, 1H), 2.60 – 2.41 (m, 2H).. HRMS, theoretical m/z: 690.0060 MH^+, observed m/z: 690.0051 MH^+.

*1.3.2. N-(3-{5-Bromo-4-[4-(trifluoromethyl)phenyl]oxazol-2-yl}-3-(3-carbamoyl-2,4-difluorophenoxy)propyl)-2-chloro-4-hydroxybenzamide (**RUP5**).*

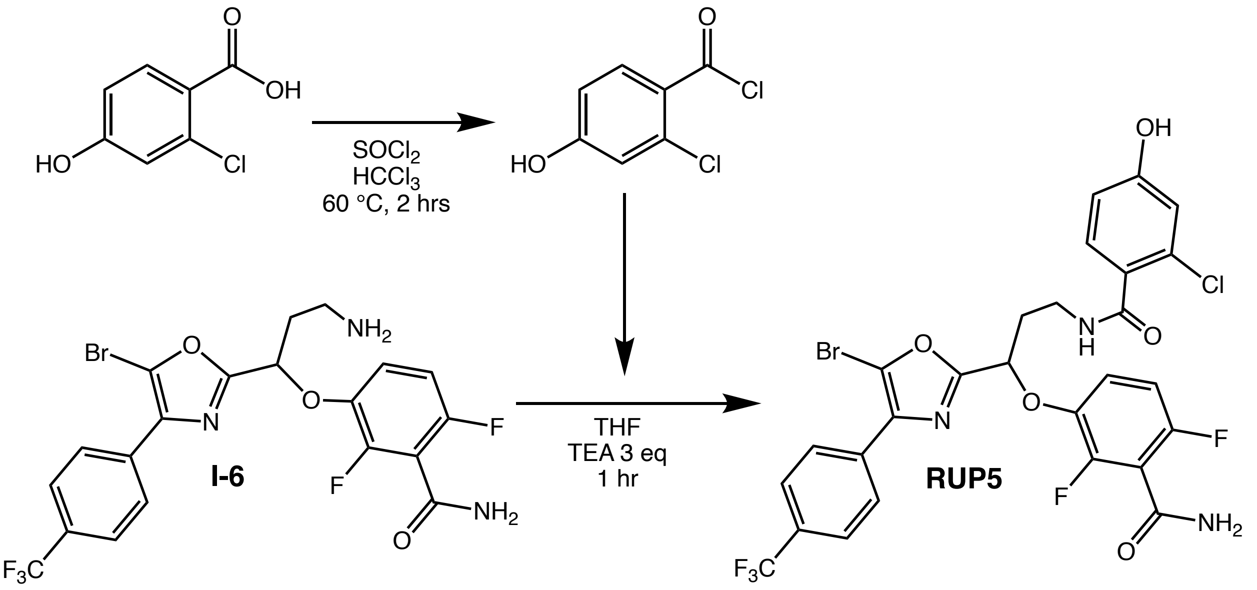

To a solution of 2-chloro-4-hydroxybenzoic acid (3.5 mg, 0.02 mmol) in chloroform (0.5 mL), $SOCl_2$ (0.002 mL, 0.02 mmol) was added. The reaction was heated at 60 °C for 2 hours. After cooling to room temperature, the solvent was removed under reduced pressure and further dried on high vacuum for 3 hours. The acid chloride residue was used in the next step without purification.

To a solution of **I-6** (19 mg, 0.04 mmol, 1 eq) in THF (2 mL), TEA (15 µL, 11 mg, 3 eq) was added followed by 2-chloro-4-dihydroxybenzoyl chloride (4.63 mg, 0.02 mmol, 1.2 eq) dissolved in chloroform (1 mL). The reaction was stirred at room temperature for 1 hour. Upon completion, the reaction was diluted with EtOAc (100 mL), washed with H_2O (35 mL), brine (35 mL), and dried over anhydrous Na_2SO_4. The solution was concentrated to give an oil, which was

purified by preparative HPLC, eluting with 1-100% CH_3CN/H_2O + 0.1% TFA. The fractions containing desired product were concentrated to afford a white solid (3.1 mg, 22.8% yield, **RUP5**). ^{1}H NMR (500 MHz, methanol-d4) δ 8.15 – 8.09 (m, 2H), 7.78 – 7.72 (m, 2H), 7.36 – 7.27 (m, 2H), 6.96 (td, J = 9.1, 1.9 Hz, 1H), 6.83 (d, J = 2.3 Hz, 1H), 6.73 (dd, J = 8.4, 2.4 Hz, 1H), 5.53 (dd, J = 8.0, 5.4 Hz, 1H), 3.74 (ddd, J = 13.8, 7.7, 6.3 Hz, 1H), 3.60 (dt, J = 13.1, 6.2 Hz, 1H), 2.50 (dddt, J = 40.1, 14.1, 7.9, 6.2 Hz, 2H). HRMS, theoretical m/z: 674.0111 MH^+, observed m/z: 674.0100 MH^+.

2. Generation of *ΔfepA, Δfiu, ΔcirA, and ΔfepC* *mutant strains of* K. pneumoniae *10031.*

Genetic deletion of iron uptake genes *fepA*, *fiu*, *cirA*, and *fepC* in *K. pneumoniae* 10031 was performed as described previously [59]. Briefly, log-phase *K. pneumoniae* 10031 cells were made electrocompetent by washing three times with ice-cold 10% glycerol. The cells were then centrifuged at 6,500 rpm for 5 minutes, the supernatant was discarded, and the cells were resuspended in the residual 10% glycerol (approximately 1 mL). 400 ng of plasmid pACBSR-hyg was then transformed into the cells by electroporation at 2500 V in a chilled 0.2-cm-gap Gene Pulser Cuvette (Bio-Rad) using a Micropulser Electroporator (Bio-Rad). The transformed cells were added to SOC media (NEB) and incubated with shaking at 30 °C for 1 hour. 100 µL was then spread onto low-salt (0.5% NaCl) LB agar plates infused with 100 µg/mL hygromycin B. These LB hygromycin plates were incubated at 30 °C for 48 hours. Successful transformations were confirmed by plasmid isolation and subsequent agarose gel electrophoresis.

Apramycin resistance cassettes flanked by flippase recognition target (FRT) sites (FRT-apramycin-FRT cassettes) were amplified from plasmid pMDIAI using the primers listed in Table S3. These primers contained sequences corresponding to 60 bp of homology either upstream (forward primer) or downstream (reverse primer) of *fepA*, *fiu*, *cirA*, or *fepC*. The design of the deletion cassette is schematically depicted in Scheme S1.

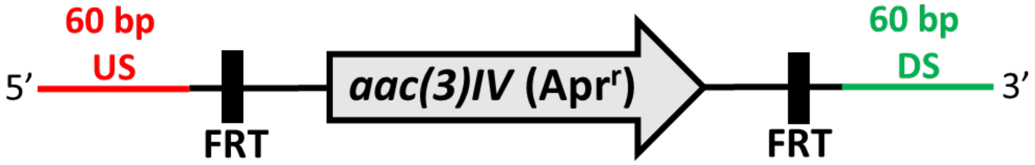

Scheme S1. Cassette design for deletion of Fe^{3+} transport genes in *K. pneumoniae*. Cassettes consisted of an *aac(3)IV* gene conferring resistance to apramycin (Apr^r) flanked by flippase recognition target (FRT) sites. The 5′ end of the cassette had 60 bp of homology to the region directly upstream (US) of the target gene, while the 3′ end had 60 bp of homology to the region directly downstream (DS) of the target gene.

1 µg of the deletion cassette was transformed by electroporation into electrocompetent *K. pneumoniae* 10031 cells containing the plasmid pACBSR-hyg. The cells were recovered in SOC media at 37 °C for 2 hours. 100 µL was then spread onto LB plates infused with 50 µg/mL apramycin. These LB apramycin plates were incubated overnight at 37 °C. Genomic DNA was then isolated from the resistant colonies using a DNeasy Ultraclean Microbial Kit (Qiagen), and the recombination of the FRT-apramycin-FRT cassette with the targeted gene was confirmed by PCR amplification using the primers listed in Table S3. The confirmed recombinants were then passaged at 43 °C overnight on LB apramycin plates to facilitate the removal of the pACBSR-hyg plasmid. Resulting colonies were then passaged overnight at 37 °C on both LB apramycin and LB hygromycin plates to confirm successful removal of pACBSR-hyg.

Electrocompetent *K. pneumoniae* 10031 cells confirmed to contain the FRT-apramycin-FRT cassette were then transformed by electroporation with plasmid pFLP-hyg. The cells were recovered in SOC media at 30 °C for 1 hour and 100 µL was then spread onto LB hygromycin plates. These plates were incubated at 30 °C for 48 hours. Resistant colonies were passaged for 24 hours at 43 °C on LB plates to remove the FRT-apramycin-FRT cassette. Resulting colonies were then passaged overnight at 37 °C on both LB and LB apramycin plates to confirm removal of the FRT-apramycin-FRT cassette. Colonies sensitive to apramycin were passaged overnight at 43 °C on LB plates to facilitate removal of the pFLP-hyg plasmid. Resulting colonies were then passaged overnight at 37

°C on both LB and LB hygromycin plates to confirm successful removal of pACBSR-hyg. Genomic DNA from colonies sensitive to both apramycin and hygromycin was purified, and deletion of the gene of interest was confirmed by PCR amplification using the primers listed in Table S3.

The plasmids pACBSR-hyg (Addgene plasmid #87830; http://n2t.net/addgene:87830; RRID:Addgene_87830) and pFLP-hyg (Addgene plasmid #87831; http://n2t.net/addgene:87831; RRID:Addgene_87831) were a gift from Pep Charusanti [59].

Supplementary References:

Supplementary references [24,58,59] are listed in the main reference list.
